# “Y no quedó nada, nada de la casa, todo salió volando” (*And there was nothing left, nothing of the house, everything flew away*): a critical medical ecological perspective on the lived experience of hurricane María in Puerto Rico

**DOI:** 10.1186/s12889-021-11847-w

**Published:** 2021-10-09

**Authors:** D. Vega Ocasio, J. G. Pérez Ramos, T. D. V. Dye

**Affiliations:** Department of Obstetrics and Gynecology, University of Rochester, School of Medicine and Dentistry, 601 Elmwood Avenue, Box 668, Rochester, NY 14642 USA

**Keywords:** Puerto Rico, Disaster, Ecosystem, Hurricane María, Social determinants, Diaspora, Medical ecology, Critical perspectives, Qualitative, Trauma

## Abstract

**Background:**

Ecological disasters create dramatic changes as man-made and natural ecosystems adapt to their effects. In 2017, Hurricanes Irma and María devastated Puerto Rico. Public focus after such traumatic ecological events often neglects pre-existing community dynamics, heterogeneity of lived experience, and complexity of decision-making in the disaster context. We intended to better understand the lived experience of this ecological trauma in communities across ecosystems in Puerto Rico and among those displaced to Florida.

**Method:**

We used the Critical Medical Ecological (CME) framework to assess the relative contribution of ecological dimensions on lived experience across community levels and time. We used qualitative methods with emic coding and etic mapping of salient constructs to the ecological model. In total, 96 people participated in 23 discussion encounters. Two people coded interviews in Spanish using Dedoose. We identified common themes in sequential order mapped to elements of the CME to approximate the participants’ temporal experience.

**Results:**

Codes applied to the period of the hurricane’s landfall, traverse, and exit were markedly distinct from the other two periods (before and after) examined in this study: the experience of the hurricane’s strike was highly personal and, at this level, reflected a mix of sociocultural, biological, and abiotic factors. After the hurricanes, social and community factors re-emerged while new risks and conditions arose that were biological (e.g., leptospirosis, no food or water) or abiotic (e.g., unusable roads/bridges, structures destroyed), but created ongoing stressors and social needs for communities. As we found, the dynamics of the social and household landscape sometimes involved the decision to leave Puerto Rico altogether, or forced people to continually face and adapt to the ongoing collapse in basic services that were only slowly and differentially restored.

**Conclusion:**

Lived experience across each stage of the hurricanes differed substantially from one another. Communities disrupted by ecological disaster are also frequently entangled within global economic and political histories and dependencies that could preclude recovery. Island nations are especially vulnerable to both climate-induced ecological change and political-economic exploitation. The ongoing health effect of the hurricane remains palpable in many communities of Puerto Rico and among the diaspora in Florida.

**Supplementary Information:**

The online version contains supplementary material available at 10.1186/s12889-021-11847-w.

## Background

Ecological disasters – increasingly more common and frequently attributed to climate change – create disbalance among physical environments, biological risks, and health care, and disrupt lives and communities that are nested within them [1]. Dramatic changes occur in human populations post-disaster as man-made and natural ecosystems adapt to their effects through recovery and regeneration, achieving homeostasis and driving toward stability or a “new normal” in both nature and society [2–4]. Public focus surrounds the occurrence of disastrous events with rescue, provision of aid, population exodus, and recovery; this focus often centers on visible effects of the disaster event itself, neglecting pre-existing community dynamics, heterogeneity of lived experience of the disaster, and the complexity of decision-making (or, lack of agency to make decisions) post-event. Often the “disaster” stretches far before and far beyond the ecological events that characterize them and adopting such a longitudinal perspective can help explain community change, while incorporating the additive impact of the event – on individuals, households, communities, and nations - itself [5].

In September of 2017, Puerto Rico was devastated by two powerful hurricanes, Irma and María, 2 weeks apart. Hurricane María, the strongest hurricane to hit Puerto Rico in more than a century, caused devastation across the archipelago [6]. The damages manifested by this hurricane destroyed telecommunication systems, energy, and water infrastructures, reshaped coastlines, damaged roads, caused mass deforestation, and destroyed homes. The toll from Hurricane María was estimated at more than 4000 people dead [4, 7, 8], and with an estimated 212,000 people or more migrating away from the archipelago [9].

The consequences of human entanglement in ecological disasters are the immediate loss of agency and the creation of vulnerability in populations shocked and living within disrupted environments [10, 11]. The events witnessed and experienced by living through the disaster itself are tragic, disruptive, surreal, and impactful, and frequently usher a post-event social and ecological disaster that creates even more disruption and suffering [12]. For instance, the 2010 earthquake in Haiti significantly destabilized natural and built environments - killing an estimated 250,000 people - but the subsequent social disaster after the earthquake, including a widespread, uncontrolled cholera epidemic, killed far more [13, 14]. The consequences of the 2010 earthquake in Haiti still reverberate in communities and in the ecologies that sustain them, with populations displaced, services disrupted, and infrastructure destroyed long after the earthquake itself [15, 16]. Similarly, the tsunami in Southeast Asia in 2004 killed 230,000 people in a short period of time, yet the destabilizing and socially destructive impacts toppled governments, caused infectious disease outbreaks, and dissolved communities long after [17–19].

**Fig. 1:**
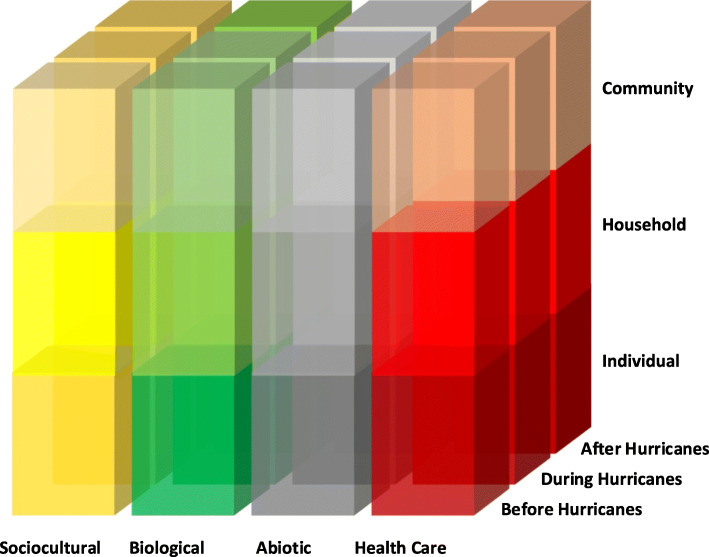
Critical Medical Ecological Multileveled Analytical Framework: Lived Experience Before, During, and After 2017 Hurricanes in Puerto Rico

We employed the Critical Medical Ecological framework [20] (Fig. 1) to examine the relative contribution of social, biological, abiotic, and health care dimensions on lived experience across communities, households, and among individuals before, during, and after the hurricane disaster in Puerto Rico. We aimed to describe the lived experience in communities across ecological zones in Puerto Rico and among Puerto Rican community members displaced to Orlando, Florida to more comprehensively understand the pre-hurricane context, to delineate the chronological stressors arising from the hurricanes, and to describe the resulting adjustments and impacts in communities. We used qualitative methods with emic (local perception) coding and etic (external) mapping of salient constructs to the ecological model to explore the dimensions of this disaster, not knowing in advance exactly what dynamics and constructs would emerge as prominent in accounting of lived experience.

## Methods

### Research team and reflexivity

The research team had several layers. This project was embedded in a larger CDC-funded initiative partnering with Puerto Rican communities and the University of Puerto Rico [21], with Dr. Dye and Dr. Pérez Ramos involved from the beginning, including a research pause to create the #p2p4PUR (people-to-people for Puerto Rico) hurricane relief initiative [22]. Drs. Dye and Pérez Ramos came to know many of the communities involved in this present study through their efforts with the CDC-funded initiative and #p2p4PUR. After the hurricanes of 2017 and with resumption of the team’s research in Puerto Rico, Dr. Vega Ocasio and Dr. Ivelisse Rivera joined Dr. Dye and Dr. Pérez Ramos in creating the protocol that governed this project. As a demonstration of equity and to deliberately avoid a hierarchical structure, the four investigators (Drs. Vega Ocasio, Rivera, Pérez Ramos, and Dye) serve as Co-Principal Investigators. All four Co-PIs were deeply familiar with Puerto Rico, with family in the islands, and directly impacted by the hurricanes. The entire field team was fluently bilingual (Spanish and English), and culturally Latin American and all coding team members were fully bilingual (Spanish-English) with Spanish as their first language. Two team members identify as men and six as women. Several team members were full-time PhD students in the University of Rochester’s Translational Biomedical Science program at the time of their involvement with this project.

The investigator team had pre-existing relationships with some communities or individuals within particular locales before starting this work. In some cases, the team networked through other contacts to reach interested organizational partners at the community level. While the team had prior experience conducting fieldwork and in coordinating the aid response throughout Puerto Rico, they did not have prior experience nor existing research relationships in Orlando, Florida, but networked through social and professional contacts to reach research partners.

Project participants knew that the field team was Puerto Rican, based in Rochester, New York, and had conducted health research in Puerto Rico before. The team members from Puerto Rico were from different parts of the country and had different diaspora experiences in the USA. The team members introduced themselves before interviews or focus groups started, sharing this background. The presence of Latin American, Caribbean, and European-American team members (in the field team and in the wider analytic team) helped us bring both *etic* and *emic* perspectives to coding and analysis.

### Study design

Our work examining the lived experience of the 2017 hurricanes among Puerto Rican residents and the diaspora used the medical ecology paradigm as a heuristic device [20, 23]. Medical ecology is particularly well-suited to help organize data and to help interpret environmental or ecological-related events in the social, biological, health care, and physical environments. The model requires that we attend to multiple levels of organization (individuals, households, communities) and across domains (biological, sociocultural, abiotic, health care), address the processes of stressors and adaptations, and evaluate different types of data. For this project, while the medical ecological paradigm helped us shape the development of an interview guide, the fieldwork was predominantly ethnographic and qualitative. This approach allowed for full expression of community voices to capture lived experience.

Participants met the inclusion criteria of a) Age 18 and over, b) self-identified as Puerto Rican, and c) were resident in Puerto Rico, the USA, or relocated to the USA since the hurricanes. We worked with local community organizations in Puerto Rico and in the USA, who serve the populations impacted by the hurricanes to help organize discussion groups and interviews (identifying venues, setting times, staffing check-in, referrals). We deliberately included a range of geographic locations to help capture variety in experience, and conducted groups and interviews in urban, mountain, coastal, and outer island locales throughout Puerto Rico. Specifically, we included the following types of municipalities and ecosystems in our study, “Urban/Metro,” “Mountain,” “Coastal,” and “Outer Island.” In the state of Florida, we concentrated groups and interviews in Orlando, to where most Puerto Ricans who left Puerto Rico for the mainland after the hurricanes relocated [24].

We worked with local community partner organizations in each ecological zone to identify potential participants in their catchment areas through flyers distributed or through social networking and word-of-mouth. In total, 96 people (31 men, 65 women) participated in a total of 23 discussion encounters (17 focus groups (four of which were in Orlando), 6 interviews). Groups were hosted in organizational or community facilities and interviews were completed in spaces determined by participants.

The ethnographic instrument used was the same for individual and group interviews, and was pretested with the project team and with people for whom Spanish was their first language. The interview guide was structured using Spradley’s “Grand Tour” and “Experience” approach [25], asking participants to describe sequentially how events unfolded before, during, and after the storms and with questions asking participants to recount their lived experience. For participants in Orlando, we asked more experiential questions regarding their decisions, circumstances, and lived experiences of relocating from Puerto Rico to Florida. Following presentation of the Information Sheet with IRB-required language, participants provided verbal informed consent to participate in the interview and also to be audio recorded. Documentation of written consent was waived since literacy is unclear, formal signing of documents in this population is typically restricted to formal legal and contractual transactions, and a signature would be the only written identifier in this study. An IRB-approved information sheet about the project and protections, with contact details for the investigators and IRB, was provided to participants. Topics and discussions often branched from the original script, following a local format of conversation. One field investigator guided the interview and the other took notes. A third attended to logistics of recording, processing paperwork, and assisting participants and moderators as needed. Refreshments were provided to all participants. Due to local institutional requirements and cost of participating, Orlando participants received $20 gift cards. All participants were provided with referral sheets in the interview that listed local and national hotlines, resources, and suggestions for counseling and assistance, and any local referrals were handled by partner organizations. The investigator teams debriefed at the end of each day, summarizing that day’s experience and planned for the next. All recordings were transcribed by people for whom Spanish was their first language, and transcripts were edited and corrected by project team members. Community participants did not review completed transcriptions. Demographic questions (place of residence, gender) were self-reported.

The average time for group interviews in Puerto Rico was 70 min, and in Orlando 90 min. The average individual interview lasted 73 min. Repeat interviews were not conducted and each participant participated only once. No one refused to participate and no one refused recording.

### Data preparation and analysis

We developed a mixed codebook, mostly using grounded procedures (emic codes) but also with several model-led codes (etic) [26]. First, the seven coders and investigators reviewed a sample of transcripts, generating a list of candidate codes arising from the data. Those codes were reviewed, merged, and consolidated. Next, codes were applied to a sample of transcripts and a final codebook assembled. The final codebook (Additional file 1) contained thematic, structural, theoretical, narrative, and descriptive codes and subcodes. The final codebook contained definitions and examples for coders to reference, and was entered into Dedoose for cloud-based coding [27]. The codebook was ordered sequentially to capture the chronological sequence of pre−/during−/post-hurricane emic codes followed by investigator-driven etic codes.

While themes were not determined in advance, codes were mapped to the component of the medical ecological model with which they best fit, to facilitate analysis within the medical ecological framework.

Coders were trained with a 10% sample of records in Dedoose until > 90% agreement was accomplished, with discrepant codes discussed and resolved. Subsequently, two coders fully coded every interview, again with discrepant codes discussed and resolved to achieve a final coded transcript. We used a range of analytic methods in Dedoose to identify frequency of codes, to pull excerpts for closer manual analysis, to examine code co-occurrences and to stratify codes by geographic unit (rural, urban, mountain, coastal, outer island, and Florida).

Analysis consisted of identifying the most commonly coded themes, and presenting codes and themes in sequential order to approximate the participants’ temporal experience. Examples are presented from each code to illustrate both typical and outlier situations to help portray the full range of lived experience. Codes were assigned domain (sociocultural, abiotic, biological, and health care) and level (individual, household, and community) values that were aggregated over the total period, and the periods before, during, and after the hurricanes. These summaries are presented as multilevel qualitative models to synthesize the types of ecological dynamics of each period.

Each partner organization provided a letter of support after reviewing the protocol, materials, and discussing the project with investigators. The University of Rochester’s Research Subjects Review Board reviewed and approved the project (RSRB00071756) as expedited, minimal risk research. All investigator and research team members completed CITI Program research, ethics, and compliance training required by the University of Rochester. Dr. Pérez Ramos and Dr. Dye also completed the required ethics training from the University of Puerto Rico earlier for the parent project.

We used the Consolidated Criteria for Reporting Qualitative Research (COREQ) checklist [28] to organize the reporting of qualitative findings.

## Results

**Fig. 2:**
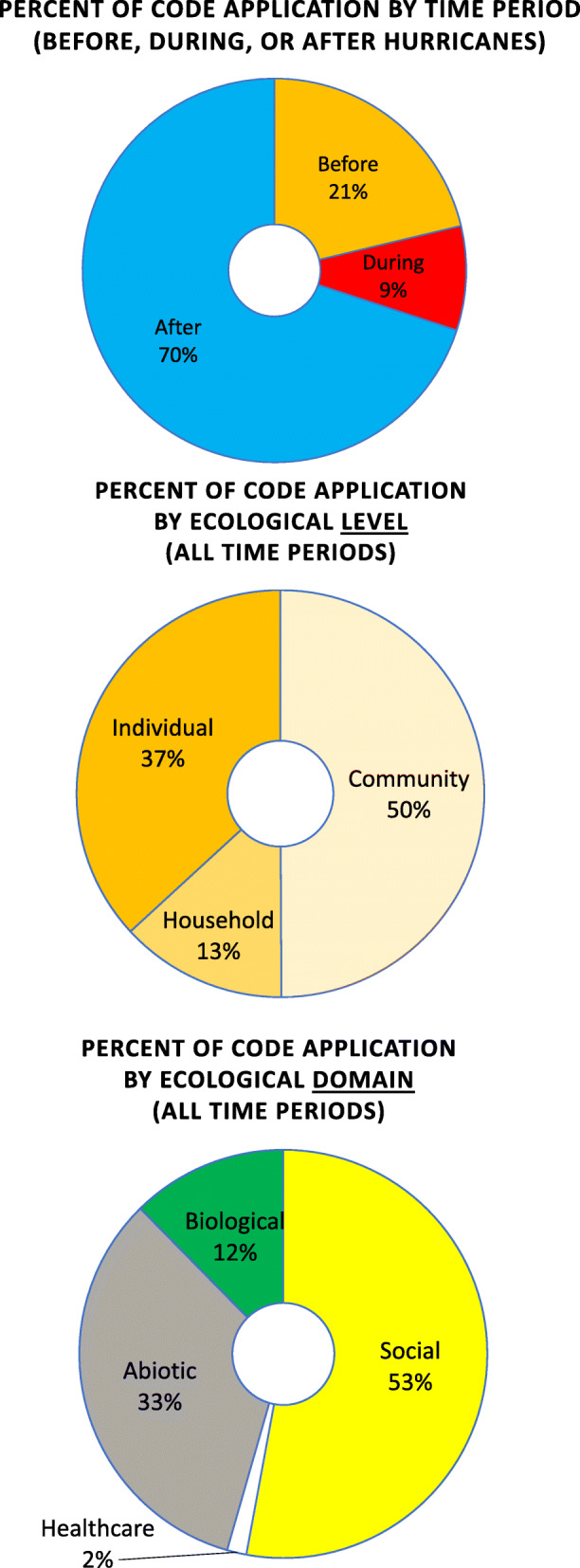
Qualitative code application by time period, domain, and level of the ecological model

As shown in Table 1, a total of 97 people from Puerto Rico (51 women and 46 men) participated in the study. Participants were recruited from different ecological zones of the archipelago of Puerto Rico and in Orlando, Florida (as a representation of a recent Puerto Rican diaspora group) to capture a range of environmental and lived experiences. Codes (see Qualitative Codes in Appendix A) were applied 3484 times to the Spanish-language transcriptions from the focus groups and interviews. In total (Fig. 2), codes from the “Before the Hurricane” period (*n* = 691) accounted for 21% of all code applications, with an additional 9% (*n* = 285) applied to the “During the Hurricane” time period, and with the majority of code applications – 70% (*n* = 2405) – applied to the “After the Hurricane” time period. An additional 122 code applications were generic or administrative, applied across all time periods. Overall, across all time periods, half of the codes applied related primarily to the “Community” domain of the ecological model (Fig. 2), followed by codes located within the “Individual” domain (37%), and “Household-related” codes (13%). Similarly, slightly more than half of the applied codes related to the “Sociocultural” domain, one-third of the codes applied related to the “Abiotic” domain, 12% to the “Biological” domain, and 2 % in the “Health Care” domain.

**Table 1:** Participants demographics by ecosystem/ region

Geographical Region	Number of Individual Interviews	Number of Focus Groups	Women	Men	Total of participants
**Coastal**	2	6	22	12	34
**Metropolitan Area**	0	3	6	3	9
**Outer Island**	3	1	5	0	5
**Rural**	1	3	12	10	22
**Florida, USA**	0	4	6	21	27
**Total**	**6**	**17**	**51**	**46**	**97**

### Part a: before the hurricane

**Fig. 3:**
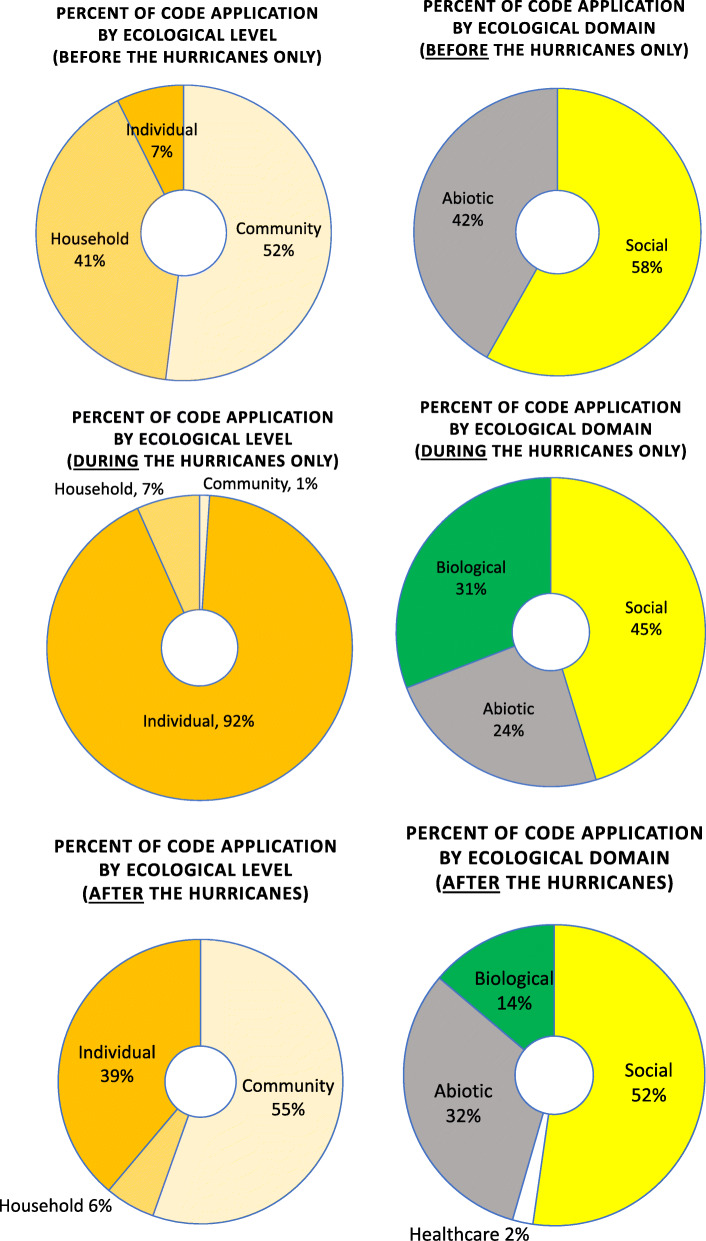
Distribution of qualitative codes by ecological level and ecological domain by period of the hurricanes (before, during, after)

Overall, half of the codes applied in reference to the period before the hurricanes related to the Community level of the ecological model (see Fig. 3), while 41% of codes applied in this period related to Household level issues, and with 7 % of the codes applied to the Individual level. All of the codes applied from the period before the hurricanes related to either Social (58%) or Abiotic (42%) factors, with no codes applied in the Biological or Health Care domains during this period.

Shown in Table 2, overall “hurricane preparation” (considered predominantly as a Household-level/ Abiotic set of codes, Fig. 3) was the most commonly-mentioned theme (particularly in the metro areas) when participants recalled the period before the hurricanes –– as people assembled food and water supplies, protected windows with shutters, and relocated to shelters or to the homes of relatives. Lack of preparation was also commonly noted, especially in coastal and rural areas, in particular a lack of prevention by government agencies. Participants in coastal and metro areas noted that flooding was already frequent common in their communities, even before María.

**Table 2:** Most commonly applied qualitative codes before Hurricane María with examples, by geographic area

	Exemplary Supportive Quotes				
Theme	Coastal	Metro	Outer Island	Rural	Diaspora
**Preparación para el huracán (Hurricane Preparation)** (Ecological Model Level: *Sociocultural domain)* *No. of mentions: 168 (4.8%*)	**“Y yo por lo menos en mi casa yo trepé casi todo en bloques porque yo vivo cerca del lago y trepé mis muebles, los zapatos de los nenes, todo yo** ***lo traté de subir.”*** *[And at least in my house I put everything in blocks because I live near a lake, and I raised the furniture, the children shoes, everything, I tried to make it higher*] *No. of mentions (%):* *63 (4.4%)*	**“Los otros huracanes que yo había vivido, que el último más grande fue George, yo era pequeña así que yo no sabía mucho de preparación de huracanes, honestamente yo tuve que meterme en google y en llamar a mi papa como para “como te preparas para un huracán” ¿verdad?. Así que básicamente hice lo mismo que habían hecho mis padres para prepararse para huracanes anteriormente porque yo no conocía de adulta huracanes.”** [*The other hurricanes that I had lived, the last biggest was George, I was little so I didn’t know much about hurricane preparation, honestly I had to google and call my dad to ask: “how do you prepare for a hurricane*” *right? So, I basically did the same thing my parents had done to prepare for hurricanes before because I didn’t know hurricanes as an adul*t”] *No. of mentions (%):* *9 (3.3%)*	**“Yo me preparé con lo básico porque yo decía “mi casa” para ese entonces porque ya yo no vivo en esa, era, es bien pequeña, entonces, este yo, al ver el último “live” de Ada Monzón, yo me asusté tanto que hice hasta bulto porque mi nene tenía seis meses para ese entonces yo decía “si pasa algo yo tengo que salir corriendo” y en Vieques es difícil porque para ese tiempo no va a haber lancha, no va a haber vuelos. Y…..Entonces hice bultos, este…mi esposo llenó más agua por si nos quedábamos sin agua y nada, nos encerramos ahí en la sala porque considerábamos que era el lugar más seguro porque era donde menos habían ventanas y como que la ventana estaba ya reforzada y pues…a esperar.”** [“*I prepared myself with the basics because I said “my house” at that time because I no longer live there, it was, it is very small, so when I saw the last live of Ada Monzón, I was so scared that I prepared a bag because my baby was six months old at that time I said “if something happens I have to run” and in Vieques it is difficult because at that time there will be no boat, there will be no flights. And ... I prepare my bags … my husband filled them with water in case we ran out of water and we locked ourselves there in the room because we considered it to be the safest place because it was where there were fewer windows and the window was already reinforced and well ... then we waited”*] *No. of mentions (%): 9(3.4%)*	**“Por lo menos nosotros en casa nos preparamos por menos con los nenes, con los medicamentos, para la fiebre, los medicamentos para las alergias de ellos, por varios días, y agua, y las tormenteras. Lo más importante para mí son los medicamentos de ellos.”** [“*At least we at home prepare for less with the babies, with the medicines, for the fever, the medicines for their allergies, for several days, and water, and the storm racks. The most important thing for me is their medications*.”] *No. of mentions: 55 (5.4%%)*	**“Llega el momento en que ya como pasa tanto tiempo, porque no esperábamos, verdad, todo el tiempo escuchábamos al gobierno que estaban preparados, pero no nos decían para cuanto tiempo estaban preparados, y entonces ahí es donde entró el pánico verdad, de que uno dice, “wow”, yo tengo gasolina o diésel para tanto tiempo” pero hasta cuándo va a durar esto, cuando ya entonces las gasolineras no tienen diésel, ni gasolina, ahí es que entonces viene lo peor.”** [“*The time comes when so much time passes, because we did not wait, right, all the time we listened to the government that they were prepared, but they did not tell us for how long they were prepared, and then that is where the panic entered the truth, that one says, “wow”, I have gasoline or diesel for so long “but how long is this going to last, when the gas stations have no diesel or gasoline, that’s when the worst comes.”]* *No. of mentions(%): 19 (3.7%)*
**Situacion Sociales (Social Circumstances)** (*Socio-cultural domain)* *No. of mentions (%): 119 (3.4%)*	**“Uno de los problemas más grandes que tiene Rincón es la salud, como tal. Es que aquí hay mucha población envejeciente, por ende, muchos de esos familiares quedan solos en sus casitas, enfermos. Aquí en Rincón, no hay una ambulancia.”** [“*One of the biggest problems Rincón has is health, as such. There is a large aging population here, therefore, many of those relatives are left alone in their houses, sick. Here in Rincon, there is no ambulance”*] *No. of mentions (%): 41 (2.9%]*	**“La limpieza y eso puede acarrear a los habitantes de estas comunidades un sin números de condiciones de salud, que sobre todo por las inundaciones, las gastritis, la dermatitis, el asma. Después del huracán vimos mucha asma, mucha conjuntivitis. Este…Hubo casos de piojos.”**[“*Cleanliness and that can lead to the inhabitants of these communities a number of health conditions, especially floods, gastritis, dermatitis, asthma. After the hurricane we saw a lot of asthma, a lot of conjunctivitis. This ... There were cases of lice”*] *No. of mentions (%): 9 (3.3%]*	**“No, no hay gasolina, no vienen trucks de comida porque los ferrys los paran completamente.”** [“*No, there is no gasoline, no food trucks come because the ferries stop them completely*.”] *No. of mentions (%): 27 (10.2%)*	**“Yo considero un problema económico el hecho de, al vivir en la región noroeste y la mayor parte de los trámites grandes se hacen en San Juan, de las agencias gubernamentales, cada vez que uno sube allá, nada más son cincuenta dólares de gasolina más el peaje, subir y bajar.”** [“*I consider an economic problem the fact that, living in the northwest region and most of the large procedures are done in San Juan, by government agencies, every time one goes up there, it is only fifty dollars of gasoline plus the toll, go up and down”].* *No. of mentions (%): 35(3.5%)*	**“Eh, el clima, el clima estaba contaminado, había muchas personas con dificultades respiratorias, infecciones en los pulmones, eh, problemas con los mosquitos, con el famoso dengue, por lo menos en donde yo vivía si.”** [“Um*, the climate, the climate was polluted, there were many people with respiratory difficulties, lung infections, uh, problems with mosquitoes, with the famous Dengue, um, at least where I lived yes”]* *No. of mentions(%): 7 (1.4%)*

From a participant who lives in a rural, coastal area:

*“Nosotros, en mi casa empezamos una preparación, pero al ir a las ferreterías no habían paneles ya y el precio que te estaban dando por un panel de un cuarto de PVC era sobre 40 dólares. El presupuesto no daba para comprar siete u ocho paneles que yo necesitaba. En mi caso yo rompí los closets y tapé las ventanas que eran de cristal, las que pude con la poca madera que tenía dentro de mi casa, sacamos y cubrimos la parte de, que es la que, como yo vivo en una lomita, la que recibe la mayor parte del impacto pero no fue suficiente. Me preparé con lo poco que tenía, forré y lo demás, compra y eso siempre los suministros para esta época pues en casa siempre se suplen, pero en el trabajo de ventanas, eso no se pudo hacer más que la mitad porque en las ferreterías la gente ya había sacado todo el panel que había y lo que quedó pues no fue suficiente. Y nosotros pues, el dinero no daba para comprar el material necesario para-- no había tormenteras y mis ventanas son de cristal. Lo poco que se pudo hacer, se hizo, lo demás pues se quedó a la intemperie y fue lo que sufrieron las consecuencias.”*

“We started a preparation at my house, but when we went to the hardware stores there were no panels anymore and the price, they were giving you for a one-quarter PVC panel was about $ 40. The budget was not enough to buy seven or eight panels that I needed. In my case I broke the closets and covered the windows that were glass, which I could with the little wood that I had inside my house, we took out and covered the part of, which is the one that, since I live on a mound, the it receives most of the impact but it was not enough. I prepared myself with what little I had, I lined and the rest, buy and that is always the supplies for this time because at home they are always supplied, but in the work of windows, that could not be done more than half because in hardware stores the People had already removed all the panel there was and what was left was not enough. And we, therefore, the money was not enough to buy the necessary material for ... there were no storm shutters and my windows are glass. The little that could be done was done, the rest was left out in the open and it was what suffered the consequences.”

From a participant who lives in a rural, coastal area:

*“Para nada, yo no me preparé. O sea, había una compra que habíamos hecho, cobramos el cheque y hacemos la compra grande del mes. Yo no vi en mi casa preparación porque “no vamos para ningún lado, nos vamos a trancar aquí y mañana es otro día, nada va a pasar””*

“Not at all, I didn’t prepare. In other words, there was a purchase that we had made, we cashed the check and we make the big purchase of the month. I did not see preparation in my house because “we are not going anywhere, we are going to lock up here and tomorrow is another day, nothing will happen”

From a participant living in a rural area:

*“Lo que pasa es que mira la gente dice “entonces, no te preparaste” pero el gobierno está preparado, nadie está preparado, ninguna agencia de gobierno se preparó, aun teniendo más conocimiento que el pueblo, ahí nadie se preparó.”*

“What happens is that when people look, they say “then, you did not prepare” but the government is prepared, nobody is prepared, no government agency was prepared, even though they have more knowledge than the people, no one was prepared there.”

**Fig. 4:**
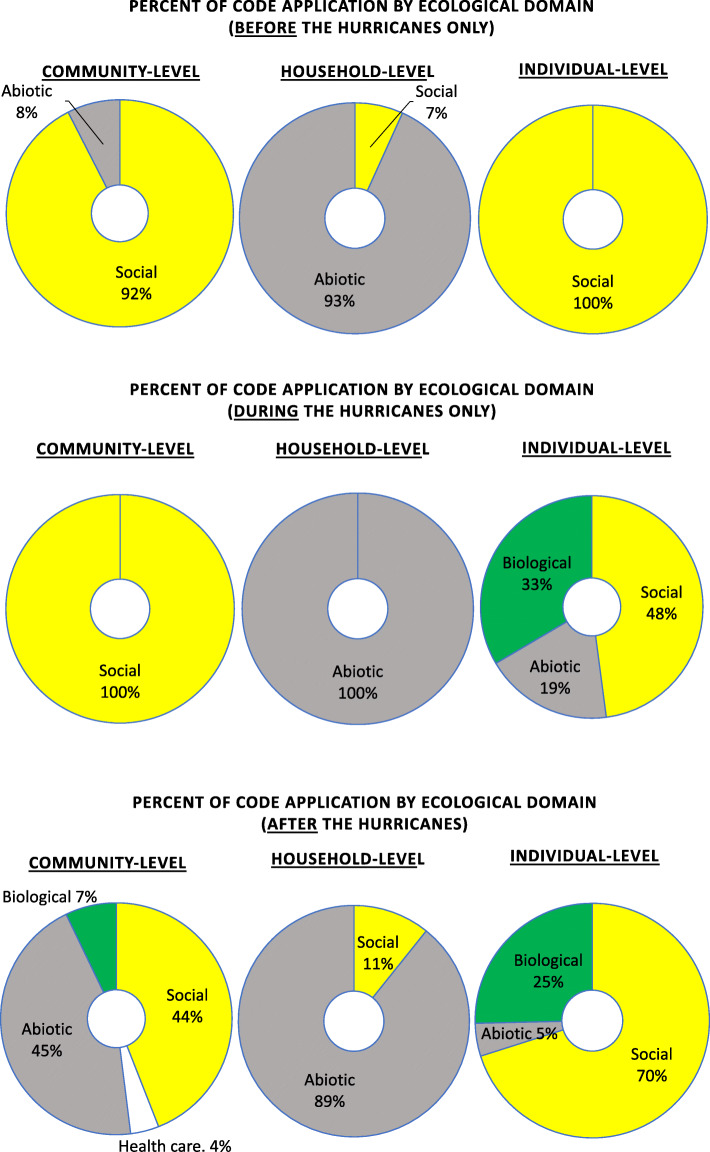
Multivariate qualitative code distribution of ecological domain by level (community, household, individual) and time (before, during, after hurricanes)

Almost all codes applied at both the Community and Individual levels in the period before the hurricanes related to sociocultural factors (Fig. 4). For instance, participants also noted challenging social circumstances that pre-existed the hurricanes such as crime, violence, and unemployment in communities, and migration from Puerto Rico to the USA. Outer island residents were particularly concerned about crime in their communities.

From a participant living in an outer island:

*“Muchas veces está bien tranquilo, pero llega una temporada que está caliente, empiezan los robos, empiezan a asaltar, la verdad? La guerra de la calle y pues estamos en ese tiempo ahora, que no puede estar por ahí mucha, mucho…. Verda? Sola… no puedes estar tan tarde por ahí y tienes que tener cuidado donde te metes.”*

“Many times, it is very calm, but there comes a season that is hot, the robberies begin, they begin to assault, right? The street war and well we are in that time now, that there cannot be around much, much…. Right? Alone… you can’t be that late out there and you have to be careful where you go.”

From a participant living in a coastal zone:

*“Aquí trataron de montar un puntito [drogas ilícitas] acá en frente y los mismos vecinos nos organizamos y le caímos arriba a los muchachos, un poquito problemático”.*

“Here they tried to set up a little point [illicit drugs] here in front and the same neighbors organized ourselves and we fell on top of the boys, a bit problematic.”

Several Orlando participants indicated that people were already leaving the island in the times before María. Participants who eventually migrated to Florida were less likely to mention their communities as tranquil before the hurricanes arrived and generally were less likely to mention preparedness-related issues.

From a participant living in Orlando:

“*…que en el área médica los médicos ya se están yendo de Puerto Rico, y hay, hay especialistas que no, que ya ni existen. O sea, básicamente. O sea que antes que pasara María, es como dijo el caballero, fue el-el-el último hachazo que nos dieron, ¿verdad?, para que Puerto Rico terminara como está.”*

“... that in the medical area the doctors are already leaving Puerto Rico, and there are, there are specialists who are not, who no longer exist. I mean, basically. In other words, before María passed, it is as the gentleman said, it was the-the-last hack that they gave us, right? So that Puerto Rico ended as it is.”

From a participant living in Orlando:

*“No hay para lo cual se sacrificaron, y como dicen en mi campo, se pelaron las pestañas. Si nosotros como padres hasta nosotros mismos los impulsamos a que busquen bienestar y yo tengo tres hijos varones. Los tres se me salieron para acá ya hace más de los últimos cuatro años. Con dolor en el alma vi a nuestros hijos partir con nuestros nietos sacándolos de-de prácticamente de debajo de nuestras sábanas, pero buscando bienestar.”*

*“*There is none for which they sacrificed themselves, and as they say in my field, they peeled their eyelashes. If we, as parents, even encourage them to seek well-being and I have three sons. The three of them left me here for more than the last four years. With pain in my soul, I saw our children leave with our grandchildren, pulling them out practically from under our sheets, but seeking well-being.”

Some participants mentioned a sense of “invincibility” before the hurricanes and that no harm would come to them, with a few noting that the comparatively low amount of damage from Hurricane Irma may have created an air of overconfidence in facing María. Also at the individual level, some participants said that they felt their faith was a tool to protect them from harm.

From a participant living in a coastal area:

*“Algunos decían que no iba a venir nada, “no, eso no viene nada”. Eso es como en otros casos que la gente decía como el cuento del lobo, viene y viene y a última hora me preparo, gasto y no viene.”*

“Some said that nothing was going to come, “no, nothing is coming.” That is like in other cases that people said like the story of the wolf, it comes and it comes and at the last minute I prepare myself, I spend and it does not come.”

From a participant living in a coastal area:

“*Entonces “Dios mío mami, entonces ¡Ay Dios! yo no puedo creer que esto va a pasar” ¿sabes?, entonces [comenzamos] rogando a Dios “vete, vete por la curvita y vete por otro lado” porque Irma lo hizo así.”*

*“*Then “My God, mom, then Oh God! I can’t believe this is going to happen” you know, then [started] praying to God “leave, leave on the curve and go the other way” because Irma did it like that.”

From a participant living in a coastal area:

“*Yo tenía miedo de lo que llegara a suceder, pero yo estaba confiado de verdad, de que, si pasaba algo, yo iba con Dios porque yo estoy en la iglesia.”*

*“*I was afraid of what would happen, but I was truly confident that, if something happened, I would go to God because I am in church.”

Despite these challenges, participants frequently describe their communities before the hurricanes as tranquil and unified. Consistent with these sentiments, some participants noted that they underestimated the level of destruction that was about to come.

From a participant living in a rural, mountainous area:

“*Aquí, como todo el mundo se conoce, entonces pues, este se unen. Entonces pues, todo es tranquilo, ¿sabes? Mayormente tranquilo. Este, no tenemos así, pues, problemas mayores de pelea, ni robo. Este, pues, podemos, pues, tratar de convivir juntos y vamos, poco a poco, uniéndonos, este, como dijo.”*

“Here, as everyone knows each other, then, well, they come together. So, everything is quiet, you know? Mostly calm. This, then, we do not have major fighting problems, nor robbery. This, then, we can, therefore, try to live together and we will, little by little, unite, this, as he said.”

From a participant living in a metro area:

“*Eh… es una, es una comunidad relativamente area tranquila que uno tiene una comunidad al lado que de hecho son una, que de hecho son los residentes originales de esa área que dura como un poquito más de ingresos, verdad, más humilde, más pobre”.*

“Eh ... it is a, it is a relatively quiet area community that one has a community next to it that in fact are one, that in fact are the original residents of that area that lasts a little more income, right, more humble, poorer.”

From a participant living in a mountainous area;

“*Tuvimos el evento de [Hurricane] Georges. Eso fue en el ‘98, ese fue fuerte. Pero anunciaban muchos fenómenos que venían, y muchas veces, pues, no llegaron, por suerte se desviaban. Había como una confianza de... digo muchos, ¿verdad? Yo entiendo que tuvimos como una confianza de que se iba a desviar. A lo mejor en muchos lugares no nos preparamos como... porque de hecho, nadie, por lo menos, de esta generación, nadie había visto algo tan fuerte como esto.”*

“We had the [Hurricane] Georges event. That was in ‘98, that was strong. But they announced many phenomena that were coming, and many times, well, they did not arrive, luckily, they deviated. There was like a confidence of ... I mean many, right? I understand that we had a kind of confidence that he was going to deviate. Maybe in many places we don’t prepare like ... because in fact, at least no one from this generation, no one had ever seen something as strong as this.”

From a participant living in a coastal area:

“*Pusimos tormenteras en la puerta del frente pero jamás y nunca pensábamos que se iba a ser como fue.”*

“We put storm shutters on the front door but never, never thought it was going to be the way it was.”

From a participant living in a rural town:

*“Pero no esperábamos el fuetazo que vino.”*

“But we did not expect the thump that came.”

### Part B: during the hurricane

Codes applied to the period of the hurricane’s landfall, traverse across Puerto Rico, and exit (see Figs. 3 and 4) were markedly distinct from the other two periods (before and after) examined in this study: the experience of the hurricane’s strike was highly personal (reflected by most codes – 92% - relating to the Individual level) and, at this level, reflecting a mix of sociocultural, biological, and abiotic factors (Fig. 4). Specifically, the predominant themes of participants from the period before the hurricanes (see Table 3) were fear and anxiety (in the Biological domain), and experiences related to the wind (in the Abiotic domain).

**Table 3:** Most commonly applied qualitative codes during Hurricane María with examples, by geographic area

	Exemplary Supportive Quotes				
Theme	Coastal	Metro	Outer Island	Rural	Diaspora
Fear and Anxiety (Biotic factors) *No. of mentions (%): 78 (2.2%)*	**“Después cuando el viento empezó había que apagamos el televisor, entonces se fue la luz. Entonces pasamos un susto bien grande porque se cayó un palo que estaba al lado de quenepa y rompió la reja de la casa y pasamos un susto bien grande. La casa se conmovió muchas veces como temblor de tierra. Yo pensaba que la casa se iba a caer del techo y entonces yo decía “¿Dios mío que yo hago aquí?”, entonces pensaba meterme al baño por si acaso pasaba algo, pero ¿y si en el baño se cierra la ventana? ¿Se tranca? Me voy a ahogar. Me va a pasar algo pensaba yo y eso.”** [“*Later when the wind started, we had to turn off the TV, then the power went out. Then we had a very big scare because a stick that was next to quenepa fell and broke the fence of the house and we had a very big scare. The house shook many times like an earthquake. I thought the house was going to fall off the roof and then I said “My God, what am I doing here?” So I thought I would go to the bathroom just in case something happened, but what if the window is closed in the bathroom? Does it lock up? I’m going to drown. Something is going to happen to me I thought and that”]* *No. of mentions (%): 48 (3.4%)*	“**Si seguía lloviendo. Había muchas teorías de ¿Por qué fue que ocurrió la inundación? La más fuerte que ha sonado es que supuestamente abrieron las puertas del Lago La Plata, las abrieron porque estaba ya en su límite y que no avisaron a nadie, ellos dicen que sí, que prendieron las alarmas de tsunami, pero nadie las escuchó y pues toda esa agua bajó; al haber tantos escombros pues se desbordó el agua y se inundaron todas estas comunidades”.** [“*If it kept raining. There were many theories of Why the flood happened. The loudest that has sounded is that they supposedly opened the gates of Lake La Plata, they opened them because it was already at its limit and that they did not warn anyone, they say yes, that they turned on the tsunami alarms, but no one heard them and then all that water went down; As there is so much rubble, the water overflowed and all these communities were flooded*.”] Santurce *No. of mentions (%) 9 (3.3%)*	**“Sí, cuando, ese momento cuando el árbol le da al carro, que ahí empezamos a escuchar, no sabíamos lo que se escuchaba, verdad? lo que era, pero se escuchaba tan fuerte. Eche yo estaba bien asustada… Yo literal me metí una Benadryl a ver si me dormía, pero los nervios eran tantos que no pude dormir. Fue bien fuerte.”** [“*Yes, when, that moment when the tree hits the car, which we started to listen to, we didn’t know what was being heard, right? what it was, but it sounded so loud. Eche I was very scared ... I literally put a Benadryl to see if I fell asleep, but the nerves were so much that I could not sleep. It was very strong.”*] Vieques *No. of mentions (%): 1(0.4%)*	**“Yo sentí miedo, cuando sentí que la casa se movía.”** [“*I felt fear, when I felt that the house was moving”]* Isabela *No. of mentions (%): 18 (1.8%)*	“**Y, cuando pasó el huracán yo estaba refugiada en una iglesia. Resulta que la iglesia es de dos niveles, y la parte de abajo tiene un colegio. Había treinta familias allí. Resulta que, en el paso del huracán, el techo de la iglesia se abrió en dos, y la iglesia se desplomó completamente la parte de arriba. O sea que la experiencia mía fue mas difícil aun, porque tuve que pasar el huracán allí en vivo, que eso fue… teníamos el agua acá arriba. Pensábamos que nos, que nos íbamos a morir, porque esa estructura empezó a temblar. Entonces, no tuvimos ropa algunos por tres días, bueno. La historia es larga.”** [“*And when the hurricane happened I was sheltering in a church. It turns out that the church is on two levels, and the lower part has a school. We had thirty families there. It turns out that, in the passage of the hurricane, the roof of the church was split in two, and the church completely collapsed on top. In other words, my experience was even more difficult, because I had to go through the hurricane there live, that was ... we had the water up here. We thought that we, that we were going to die, because that structure began to shake. So, we didn’t have any clothes for three days, well. The story is long.”]* *No. of mentions(%): 2(0.4%)*
Stories about the wind (Abiotic factors) *No. of mentions (%): 49 (1.4%)*	**“Y no quedó nada, nada de la casa, se llevó la nevera, todo lo que estaba alrededor de la pecera, todo salió volando. Como es como un laberinto, el viento daba regresaba y nos batía porque fue como una máquina de moler y allí fue destrozando todas las casas. Después tú notabas que se terminó, ya en mi casa no estaban los vientos, pero yo veía el resto de viento destrozando las casas. No hubo virazón como antes. En mi casa explotaron las ventanas y se metieron hacia adentro.”** [“*And there was nothing left, nothing from the house, he took the refrigerator, everything that was around the fish tank, everything flew away. As it is like a labyrinth, the wind would return and beat us because it was like a grinding machine and there it was destroying all the houses. Later you noticed that it was over, the winds were no longer in my house, but I saw the rest of the wind destroying the houses. There was no turn as before. In my house the windows exploded, and they went inside”]* *No. of mentions (%): 18 (1.3%)*	**“Bueno, en mi caso yo estuve agarrando ventanas para que no se abrieran y mi hija, agarraba otra. En mi casa hay una puerta que es como de cristal y a mi mamá le daba miedo que se fuera a salir, así que ella la abrió un poco y se quedó velándola desde lejos que no se fuera a volar. Ella entró en crisis porque el televisor estaba enfrente a esa puerta de cristal, y empezó, “¡Pero mueve la puerta!”, y yo la miro y yo, “Ahora no es la hora, lo siento por ti, sí se va el televisor bye, bye, tv”, pero gracias a Dios no le pasó nada. Si, nos quedamos agarrando las puertas”** [“*Well, in my case I was grabbing windows so they wouldn’t open and my daughter grabbed another. In my house there is a door that is like glass and my mother was afraid that it would leave, so she opened it a little and kept watching from afar that it would not fly away. She went into a crisis because the TV was in front of that glass door, and she started, “But move the door!”, And I look at her and I, “Now is not the time, I feel sorry for you, yes the TV bye, bye, tv”, but thank God nothing happened to him. Yes, we were holding the doors.”]* *No. of mentions (%): 7(2.5%)*	“**El viento era… Era una furia, era… o sea yo pasé Georges, yo me acuerdo de Hugo. Pero realmente esto fue, esto no se… ni… esto no se compara. No se compara.”** [T*he wind was ... It was a fury, it was ... that is, I passed Georges, I remember Hugo. But really this was, this I don’t know… nor… this doesn’t compare. It does not compare.”]* *No. of mentions (%): 3 (1.1%)*	**“En mi casa el viento se llevó todas las tormenteras que daban para atrás... se las llevó todas, y las tiró para un lado y al otro día amanecieron en la dirección completamente opuesta de donde salieron”.** [“*In my house the wind carried away all the thunderstorms that were giving backwards ... it took them all, and threw them to one side and the next day they woke up in the completely opposite direction from which they came”*] *No. of mentions (%): 17 (1.7%)*	**“Una ventana que no está para afuera, está dentro de la casa, da para la sala, de un cuarto para la sala, hizo, empezó a sonar así a la una que todavía no había nada y eso me levantó. Después de eso, nada, empiezo con mi esposo a escuchar la radio, y todo y eso era un monstruo sonando cuando empezó el pleno, yo creo que eran como las tres y media de la mañana y eso era un monstruo, realmente tú escuchabas un monstruo, lo que tú escuchabas por las ventanas eso era un monstruo que estaba afuera.”** [“*A window that is not facing the outside, is inside the house, it opens up to the living room, from a room to the living room, it did, it started to sound like that at one o’clock that there was still nothing and that lifted me. After that, nothing, I started with my husband listening to the radio, and everything and that was a monster sounding when the plenary session began, I think it was like three thirty in the morning if more or less there) And that was a monster, really you heard a monster, what you heard through the windows that was a monster that was outside”]* *No. of mentions (%): 4 (0.8%)*

Participants described Hurricane María’s experience mostly as one filled with fear and anxiety (see Table 3), most prevalent in those living in coastal areas followed by those living in rural areas. The main causes of fear and anxiety were described as a result of a rapidly deteriorating built and natural environment during the storm, combined with not knowing what was happening due to communications black-outs (telephone services, internet, and social media). This uncertainty, combined with the strong winds and the darkness, made individuals feel fearful.

From one rural, coastal participant:

“*Era como si fuera eterno. No acababa. Nos metimos en la iglesia como desde las siete de la noche hasta el otro día que terminó el huracán. Y era un miedo horrible, y la puerta comenzó a hamaquearse, la de enfrente, y estábamos en esa de que en cualquier momento se fuera a despegar. Y el agua entrando por las ventanas*.”

“It was as if it was eternal. It didn’t end. We went into church from about seven at night until the other day the hurricane ended. And it was a horrible fear, and the door began to swing, the one in front, and we were in the position that at any moment it would take off. And the water entering through the windows.”

From a participant living in a rural area:

*“Pero a mí me dio ansiedad cuando no había ningún tipo de comunicación. No había forma de... no había celulares, no había, este, televisión, no había señal. Este, bueno, tampoco tenía luz, no había”*

*“*But it gave me anxiety when there was any type of communication. There was no way to … there were no cellphones, no television, there was no signal. This, well, we had no electricity either, there wasn’t”

From a rural, mountainous area:

“*Entonces, pues, según iba aumentando la situación, nos íbamos poniendo nerviosos, ¿verdad? Porque es algo que no… desconocemos. Entonces, este, cuando nosotros nos reunimos todos aquí la familia, pues, dejaron la puerta del frente abierta, verdad, y cuando comenzó esa tormenta... pues….digo yo, para mí, yo nunca había visto algo así tan fuerte. Porque cuando Georges, esto, ese fue de noche, y me dio miedo porque la casa como que vibraba y era un zumbido terrible. Entonces, pues, yo pensé que eso podía suceder. Pero dejaron la puerta abierta y todos ahí, mirando por la puerta porque el viento se metió por aquí, por aquí al frente. Y era como un humo, el agua se convertía como en un humo, que tú no veías nada. Solamente un zumbido, verdad, como un bramido de algo...”*

“So, then, as the situation increased, we were getting nervous, right? Because it is something that we are not… unknown. So, this, when we all gathered here, the family, well, they left the front door open, right, and when that storm started ... well ... I say, for me, I had never seen something like this so strong. Because when Georges, this, that was at night, and it scared me because the house kind of vibrated and was a terrible hum. So, well, I thought that could happen. But they left the door open and everyone there, looking out the door because the wind blew in here, this way out front. And it was like a smoke, the water became like a smoke, that you didn’t see anything. Just a buzz, right, like a bellow of something ...”

From one participant – who had relocated to Orlando:

“*Cuando pasó el huracán yo estaba refugiada en una iglesia. Resulta que la iglesia es de dos niveles, y la parte de abajo tiene un colegio. Habíamos treinta familias allí. Resulta que, en el paso del huracán, el techo de la iglesia se abrió en dos, y la iglesia se desplomó completamente la parte de arriba. O sea que la experiencia mía fue mas difícil aun, porque tuve que pasar el huracán allí en vivo, que eso fue… teníamos el agua acá arriba. Pensábamos que nos, que nos íbamos a morir, porque esa estructura empezó a temblar. Entonces, no tuvimos ropa algunos por tres días, bueno. La historia es larga. La cosa es, que, (pausa) (empieza a reír) …me fui, se me fue el hilo.”*

“And when the hurricane passed I was taking refuge in a church. It turns out that the church is on two levels, and the lower part has a school. There were 30 families there. It turns out that, in the wake of the hurricane, the roof of the church split in two, and the church completely collapsed on top. So my experience was even more difficult, because I had to pass the hurricane there live, that that was ... we had the water up here. We thought that we, that we were going to die, because that structure began to shake. So we didn’t have some clothes for 3 days, well. The story is long. The thing is, that (pause) (starts to laugh) ... I left, the thread ran out.”

From a participant living in a rural area:

“*Eso le da pánico a cualquiera, al que no le dio pánico allí, le dio cualquier otra cosa.*”

“That makes anyone panic, who didn’t panic there, gave anything else.”

Mentioned previously, the vast majority of codes applied to the period of the hurricane strike itself related to Individual-level experience, which differed significantly from the pre-hurricane Individual-level experience in that the Biological and Abiotic domains are much more commonly mentioned during the lived experience of the storm (Fig. 3). Among the range of lived experience at the individual and abiotic domain, recollections of the “wind” generated by the storm are notable as the second most commonly applied code to this period. During the hurricane, many participants – especially those from the coastal and rural areas – narrated wind-related experiences: the strong winds felt like tremors, caused damage to their and their neighbors’ homes, and required creation of physical barriers to reinforce doors and windows to prevent damage significant injuries.

From a participant living in an outer island:

*“Tú sentías, la casa que quería levantar la tierra, tu sentías la casa temblaba. Como que se metió por debajo…”*

“You felt the house that wanted to raise the earth, you felt the house trembled. It kind of got underneath…”

From a participant living in a rural town:

“*Eso es angustioso, es preocupante porque, pues, nosotros sabíamos, pues, que estábamos ahí resguardados, que con esto, de verdad que ahí ya no había nada seguro…”*

“That is distressing, it is worrying because, well, we knew, well, that we were there sheltered, that with this, there really was nothing safe there ...“

From a participant living in a coastal area:

*“Eso fue horrible, y el ruido de las cosas cuando se van yendo, eso te da…te lleva a un estado de desesperación, que….. horrible. Lo que más era el ruido, el ruido es lo que te ponía, porque tú estás seguro, sin ver; todo cerrado, todo tapado, eso es lo que más te desespera, ¿sabes? de que como estará afuera, ya el momento en el que tú sales, te da una depresión horrible, porque eso es una depresión, tú ves todo en el piso, los postes en el piso, no hay paso por ningún lado.”*

“That was horrible, and the noise of things when they are leaving, that gives you … it takes you to a state of despair, which … horrible. The most was the noise, the noise is what made you, because you are safe, without seeing; everything closed, everything covered, that’s what makes you desperate the most, you know? How things will be outside, the moment you leave, it gives you a horrible depression, because that is a depression, you see everything on the floor, the [utility] posts on the floor, there is no passage anywhere.”

From a participant living in a metro area:

*“Durante del huracán. Este…nosotras nos levantamos y mi pareja me dice, “Se va a meter el agua, ¿sabes?, no hay opción, el agua se va a meter -y venía con bastante fuerza- recoge, todo lo que puedas, sube todo lo que puedas que nos vamos.”*

“During the hurricane. Um …. We got up and my partner tells me: “The water is going to get in, you know? There is no option, the water is going to get in and it came with enough force … “pick up everything you can, raise up everything you can, we are leaving””

From a participant living on an outer island:

“*Si, cuando, ese momento cuando el árbol le da al carro, que ahí empezamos a escuchar, no sabíamos lo que se escuchaba, verdad? lo que era, pero se escuchaba tan fuerte. Eche yo estaba bien asustada… Yo literal me metí una Benadryl a ver si me dormía, pero los nervios eran tantos que no pude dormir. Fue bien fuerte.”*

“Yes, when, that moment when the tree hits the car, which we began to hear there, we didn’t know what we heard, right? what it was, but it sounded so loud. Eche I was very scared ... I literally put a Benadryl to see if I fell asleep, but the nerves were so many that I could not sleep. It was very strong.”

From a participant living in the metro San Juan area:

“*Ero[a] una cosa que ahí me-me llamó mucho la atención que el oído—incluso en algunos videos que ahora podido descubrir que están en YouTube de gente que toma videos, en aquel momento era—había un sonido bien particular que se estaba dando, que yo decía: “De dónde r-rayos salió ese sonido?” Porque era como si estuviera pasando por, el viento por algún tipo de estructura que le estuviera dándole un silbido, un sonido bien particular y era un sonido de fondo, como, como bien fuerte d-del viento, como si algo estuviera rugiendo. Y entonces e-ese sonido ahí yo no lo había sentido nunca en ninguno, en ninguno otro de los, de los eventos y era como un sonido que-que aumentaba y disminuía, pero que siempre estaba ahí presente; y ese sonido yo-yo lo he podido ver en… lo he oído en uno d-d-de los videos que han puesto de la gente que ha tomado de María que me di cuenta de repente que no era particular de ahí de donde yo estaba. Ese sonido se estaba dando en todo Puerto Rico, tú sabe, y también suena como si fuera un jet, e-e-el motor de un jet de un avión que estuviera ahí prendido y hace un ruido bien fuerte que se queda ahí, constante y es donde ahí veo: “Uy, esto… es otra cosa”*

“It was one thing that caught my attention there - even in some videos that I have now been able to discover on YouTube of people who take videos, at that time it was - there was a very particular sound that was taking place, that I said: “Where did the sound come from? Because it was as if it were passing by, the wind through some kind of structure that was giving it a whistle, a very particular sound and it was a background sound, like, as strong d-from the wind, as if something was roaring. And then e-that sound there I had never felt in any, in any other, of the events and it was like a sound that-that increased and decreased, but that was always there; and that I-I sound I could see it in ... I heard it in one of the videos that they have put up of the people that they have taken from Maria that I suddenly realized that I was not particular from where I was. That sound was happening all over Puerto Rico, you know, and it also sounds like it was a jet, ee-the jet engine of an airplane that was on there and makes a very loud noise that stays there, constant and is where there I see: “oops, this ... is something else”.”

### Part C: after the hurricane

Shown in Fig. 3, narratives after the hurricane returned to a focus – overall – on community-level factors, as was the case before the hurricanes. The pre-hurricane focus on household factors (largely, preparation) was replaced after the hurricanes with a focus on individuals, largely around factors in the social and biological domains (see Fig. 4). The predominant themes of participants from the period after the hurricanes (see Table 4) were collapse of basic services, help and response, community health, and relocation to the USA.

**Table 4:** Most commonly applied qualitative codes after Hurricane María with examples, by geographic area

	Exemplary Supportive Quotes				
Theme	Coastal	Metro	Outer Island	Rural	Diaspora
Collapse of basic services (Abiotic factors) *No. of mentions (%): 345 (9.9%)*	**“La mayoría de la gente de aquí, de Playa, Verde Mar, toda esta área, cayó en una depresión bien mala porque tú sabes lo que era tú hacer una fila para que te dieran un platito de comida que había gente que no tenía en la casa una botella de agua. Pasamos esta situación bien mala, la pasamos brutal, la pasamos bien brutal. Había veces que hacías fila y lo que te daban era un solo plato de comida y hasta peleaban por la comida teniendo tú otra familia en tu casa. No, se pasó! Lo que nosotros vivimos aquí, yo creo que nunca nosotros habíamos pensado vivir una cosa igual. Sin tener un plato de comida o una botella de agua, todo eso lo pasamos.”** [“*Most of the people here, from Playa, Verde Mar, this whole area, fell into a very bad depression because you know what it was like to stand in line to get a plate of food that there were people who did not have in the home a bottle of water. We went through this very bad situation, we had a brutal time, we had a very brutal time. There were times when you stood in line and what they gave you was a single plate of food and they even fought over food with you having another family in your house. No, it happened! What we live here, I believe that we had never thought to live something like it. Without having a plate of food or a bottle of water, we had all that*.”] *No. of mentions (%): 151 (10.7%)*	**“La gasolina, entonces comenzó todo eso que fue terrible, terrible; había que venir para acá [San Juan], y entonces las filas eran kilométricas para regresarme a Fajardo.”** [“G*asoline, then all that began that was terrible, terrible; I had to come here [San Juan], and then the lines were kilometers long to return”]* *No. of mentions (%): 24 (8.7%)*	**“Nosotros estuvimos sin luz diantres hace como dos meses [Marzo 2018] vino la luz verdad? y porque hay plantas Diesel”** [“*We were without power for about two months [March 2018] the power came right? and because there are diesel plants”*] *No. of mentions (%) 23 (8.6%)*	**“Yo digo que lo más difícil para esta comunidad fue el agua y la luz, porque nosotros estuvimos seis meses sin agua, porque ahora por lo menos la tenemos dos días sí y un día no, pero estuvimos seis meses sin agua y siete sin luz.”** [“*I say that the most difficult thing for this community was water and electricity, because we spent six months without water, because now we have at least two days and one day no, but we spent six months without water and seven without electricity*.”] *No. of mentions (%): 10 (10.0%)*	**“Luego del huracán obviamente, pues el factor, verdad, que enfrentamos, este, la comunidad pues se fue abajo, los servicios pésimos, en cuanto a recogido de escombro y demás, este, sin luz por más de seis meses, desde el huracán, este, Irma, el servicio de agua pésimo también, este, las necesidades básicas, pues día a día tenemos que luchar contra eso, este, levantarnos, dormíamos apenas una hora, levantarnos para ir a las gasolineras a buscar diésel, gasolina, eh, suministros de comida, este, agua potable, en mi caso…”**[“*After the hurricane obviously, because the factor, right, that we faced, this, the community because it went down, the terrible services, in terms of collection of rubble and others, this, without light for more than six months, since the hurricane, This, Irma, the water service is terrible too, this, the basic needs, because day by day we have to fight against that, this, get up, we slept for just an hour, get up to go to the gas stations to get diesel, gasoline, eh, food supplies, this, drinking water, in my case ...”]* *No. of mentions (%): 46 (8.9%)*
Health-related problems (combined with mental health) (Biotic domain) *No. of mentions (%): 133 (3.8%) and 80 (2.3%)*	**“No hubo un diabético que no se descontroló, o sea por falta de la insulina, por las pastillas que no aparecían, nadie se tomaba los medicamentos porque tenían muchas cosas más importantes que resolver. Cuando vinieron los hospitales que pudieron abrir, estaban saturados porque tenían aquel que se metió al campo a bregar, y a sacar palo, con las patas podridas; porque no tenía el control y la diabetes es una cosa, y yo te hablo por mí, que soy diabético.”** [“*There was not a diabetic who did not get out of control, that is, because of a lack of insulin, because of the pills that did not appear, nobody took the medications because they had many more important things to solve. When the hospitals they were able to open came, they were saturated because they had the one who went into the fields to struggle, and to take out a stick, with rotten legs; because I was not in control and diabetes is one thing, and I speak to you for myself, that I am diabetic.”*] *No. of mentions (%): 33.0 (2.3%) and 19 (1.3%)*	**“Pero tú lo veías en la gente, tú hablabas con la gente y empezaba a llorar, hablabas con la gente y empezaban a llorar… este, y en la fila a los puertorriqueños nos gusta hablar mucho y para todo había que hacer una fila, para todo había que hacer, así que todo el mundo hablaba con todo el mundo y tu seguías escuchando el problema del otro y del otro y del otro y del otro y del otro.”** [*But you saw it in the people, you talked to the people and they started crying, you talked to the people and they started crying ... this one, and in line we Puerto Ricans like to talk a lot and for everything you had to line up, to everything had to be done, so everyone talked to everyone and you kept listening to the problem of the other and the other and the other and the other and the other.”]* *No. of mentions (%): 10 (3.6%) and 9 (3.3%)*	**“La desolación cuando pasa el huracán… eso sí que tú caías en una depresión. Se deprime cualquiera. No sabes lo que es mirar para el mar, no ver una lancha, no ver un barco, no ver un avión, no ver comunicaciones. Ay no… horrible”** [“T*he desolation when the hurricane passes ... that you did fall into a depression. Anyone gets depressed. You don’t know what it is to look at the sea, not to see a boat, not to see a ship, not to see an airplane, not to see communications. Oh no ... Horrible.”]* *No. of mentions (%): 16 (6.0%) and 9.0 (3.4%)*	**“Suicidios que están sucediendo, todavía yo creo que eso debe continuarse ante la emergencia, porque la emergencia pasó, pero las situaciones que arrastra la emergencia no han pasado. Y entonces yo creo que las respuestas tanto del gobierno especialmente en cuanto brindar los psicólogos, las facilidades, pues debe hacerse en continuo, no terminar porque a veces como pasó la emergencia, igual como cuando indicamos anteriormente, pasó, nos ayudamos, pasó la emergencia, se terminó.”** [“*Suicides that are happening, I still believe that this should continue in the face of the emergency, because the emergency has passed, but the situations that the emergency drags on have not passed. And then I believe that the responses both from the government, especially in terms of providing psychologists, facilities, as it must be done continuously, not ending because sometimes as the emergency happened, just as when we indicated above, it happened, we helped each other, the emergency happened, was over.”*] *No. of mentions (%): 51 (5.0%) and 33 (3.3%)*	**“En mi caso, mi suegra era paciente asmática, siempre andaba con su bultito y en diciembre comenzó a enfermarse y entonces este…cae hospitalizada, queda hospitalizada, pero al lado de ella, fallece una señora en el mismo cuarto, y tardaron en buscar a esa persona fallecida sobre ocho horas, y ella entró en un estado de ansiedad, que pidió que le dieran de alta, aunque ya había comenzado el tratamiento de antibiótico, se va a la casa y al otro día regresa y luego de ahí falleció. (esas bacterias) O sea que….Entendemos que es la raíz, ¿verdad? a consecuencia del mismo huracán, porque una persona cuando fallece no va estar tantas horas en el cuarto, pero todo es a raíz y a consecuencia de tantas muertes que hubo, que no son directamente del huracán, del día del huracán, pero eso semanas y meses, es la consecuencia de…”** [*“In my case, my mother-in-law was an asthmatic patient, she always walked with her lump and in December she began to get sick and then this ... she falls hospitalized, is hospitalized, but next to her, a woman dies in the same room, and they took time to look for That person died about eight hours, and she entered a state of anxiety, which asked to be discharged, although she had already started the antibiotic treatment, she goes home and the next day she returns and then she died. (those bacteria) So… We understand that it is the root, right? As a result of the hurricane itself, because a person when he dies will not spend so many hours in the room, but everything is as a result and as a result of so many deaths that there were, which are not directly from the hurricane, from the day of the hurricane, but weeks and months, is the consequence of…”*] *No. of mentions (%): 23 (4.5%) and 10 (1.9%)*
Help and Response (*Socio-cultural domain)* *No. of mentions (%): 147 (4.2%)*	**“Este, el vecino no nos dio un cordón, ni luz, ni agua, ni hielo, no dio nada. Y entonces yo digo, ¿a dónde está la humanidad?, ¿dónde está el amor? Decía yo, de Dios. Que nadie comparte con uno porque no es conveniente. Y entonces, hay otra cosa más. Que la comunidad no ayudaba a nadie y yo ya decía yo del otro lado pero que de todo esto yo vi, de todo esto yo noté que el gobierno estaba flojo. Porque cuando ellos necesitan que estemos nosotros, el gobierno nos ayudó porque era conveniente, la ayuda no llegaba adecuadamente porque era conveniente, usaban obreros como era conveniente, porque le pusieron los toldos, pero nunca hubo ayuda de verdad, de un zinc o un cemento para terminar tu casa. Ese es el fallo que hubo en FEMA, como hubo en FEMA y el gobierno hubo un fallo sobre eso ¿me entiendes? La gente hablaba del gobierno mal.”** [*“This, the neighbor did not give us a cord, no electricity, no water, or ice, he did not give us anything. And then I say, where is humanity? Where is love? I said, of God. That nobody shares with you because it is not convenient. And then there is one more thing. That the community did not help anyone and I already said I was on the other side but that I saw all this, all this I noticed that the government was lazy. Because when they need us to be there, the government helped us because it was convenient, the aid did not arrive properly because it was convenient, they used workers as it was convenient, because they put up the awnings, but there was never real help, of a zinc or a cement to finish your house. That is the ruling that there was in FEMA, as there was in FEMA and the government there was a ruling on that, do you understand me? People spoke of bad government”*] *No. of mentions (%): 7 (4.9%)*	**“Aunque tengamos frustraciones y otra cantidad de cosas por otras cosas que pasan en el país, ¿no? Hay que tomar en cuenta que Puerto Rico económicamente pues no está bien, que quizás no recibimos todas las ayudas que hubiéramos esperado sobre todo cuando comparamos ayudas que FEMA le dio, el gobierno federal le dio a otros estados, yo creo que a nosotros nos dio bien poco cuando yo creo que más del 80% de la isla quedó destruida. 100% sin luz de la isla.”** *[“Although we have frustrations and other things due to other things that happen in the country, right? We must take into account that Puerto Rico economically is not well, that perhaps we did not receive all the aid that we would have expected, especially when we compare aid that FEMA gave it, the federal government gave other states, I think it gave us very little when I believe that more than 80% of the island was destroyed. 100% without island light.*”] *No. of mentions (%): 7 (2.5%)*	**“Pues yo encuentro que aquí no se respondió como se debió responder. Aquí se pudo haber hecho muchas cosas que no se hicieron desde, desde el “mayor” como digo yo, se bloqueó de tal manera que, que no se movió, o sea no, no daba instrucciones, no daba órdenes, el que las daba era el de manejo de emergencias… Este, yo tuve un poste tirado frente a mi casa como una semana, y mi casa es principal. O sea, yo vivo en una calle principal frente al muelle, una semana expuesta allí, sino te cuento más, este, te estaría mintiendo, pero más de una semana, pero era tan y tan pesado que los postes, se cayó encima del establecimiento de al frente, tú sabes.”** [*“Well, I find that here it was not answered as it should have been answered. Here many things could have been done that have not been done since, from the “older” as I say, it was blocked in such a way that, that it did not move, that is, no, it did not give instructions, did not give orders, the one who gave them It was the emergency management ... This, I had a pole thrown in front of my house for about a week, and my house is main. I mean, I live on a main street in front of the pier, a week exposed there, if not I’ll tell you more, this one, I would be lying to you, but more than a week, but it was so and so heavy that the poles fell on top of the establishment from the front, you know”*] *No. of mentions (%): 13 (4.9%)*	**“En el caso del barrio, la calle principal, donde todo, es una calle que da la vuelta completa. La calle principal estaba intransitable y había una persona muerta en el barrio, que necesitaban sacarla. Y los que estaban en el barrio, se unieron para abrir brechas y que pudiera pasar la guagua de la funeraria, para que vinieran a buscar el cuerpo. Ellos estuvieron dándole como dos o tres días, o algo así, pegando con todo lo que encontraban. Pero todos los del barrio, allá abajo del bregando con eso, porque la… no había forma. El municipio no ayudó para abrir caminos, ni nada. Que estaban nosotros aquí, esto era como una islita, separado de todo. Este….Nosotros tenemos que resolvernos. Si queríamos salir, teníamos que nosotros luchar para salir nosotros.”** [“*In the case of the neighborhood, the main street, where everything, is a street that goes around completely. The main street was impassable and there was a dead person in the neighborhood, who needed to be removed. And those who were in the neighborhood, united to open breaches so that the bus from the funeral home could pass, so that they could come to look for the body. They were giving it like two or three days, or something like that, hitting with everything they found. But everyone in the neighborhood, down there from the* *struggling with that, because the… there was no way. The municipality did not help to open roads, or anything. That we were here, this was like an island, separated from everything. This .... We have to solve ourselves. If we wanted to get out, we had to fight to get out ourselves.”*] *No. of mentions (%): 47 (4.6%)*	**“El paso de la tormenta, cuando pasó la tormenta, nosotros nos sentimos solos, nadie se acercada donde nosotros, [hablante masculino 1: nadie.] nadie nos llevó una botella de agua, nadie nos llevó un plato de comida, teníamos un vecino que se pasaba gritando porque nadie se acercaba a él también. Era un ancianito de noventa y pico de años, como noventa y cuatro años.”** [“*The passing of the storm, when the storm passed, we felt alone, nobody approached where we, Nobody brought us a bottle of water, nobody brought us a plate of food, [male speaker 1: absolutely nothing] we had a neighbor who kept screaming because no one approached him too. He was an old man of ninety-odd years, like ninety-four years old.”*] *No. of mentions (%): 10 (1.9%)*
Relocating to the USA (*Socio-cultural domain)* *No. of mentions (%): 79 (2.3%)*	**“No puede trabajar. Pero la esposa, la esposa si trabaja, trabajaba aquí, pues al irse de aquí pues tuvo que dejar el trabajo, ahora están allá, que no están bien porque están metidos en la casa de cuñado, y hay un dicho que dice “el muerto a los tres días apesta”** [“*It cannot work. But the wife, the wife does work, she worked here, because when she left here she had to leave work, now they are there, they are not well because they are in the brother-in-law’s house, and there is a saying that says “the dead man after three days it sucks* “] *No. of mentions (%): 20 (1.4%)*	***“El número oficial es como 100 mil personas [se fueron de la isla] pero quizá sea muy bajo, por ejemplo ahorita les conté que la urbanización donde reside mi papá hay 33 casas. Seis familias se fueron. De 33.”*** [*“The official number is like 100,000 people [left the island] but it may be very low, for example I told you just now that the urbanization where my father lives there are 33 houses. Six families left. From 33″*] *No. of mentions (%): 3 (1.1%)*	**“Por no me fui, pues porque, porque tenía un trabajo y tenía mi esposo un trabajo, pero de que me quería ir, todos los días lo decía, “yo me voy, yo no quiero estar pasando por esto”. Porque yo digo a veces, verdad, la infraestructura aquí está abismada. Tú vas a otros países y tienen estos mismos aprietos, pero la mejoría es más rápido, más rápido. Yo a veces digo, acá es como que, no sé si es que vivimos en un área muy estratégica para los huracanes, y nos parte por el medio, pero es bien difícil, ¿cómo subir? Que si es difícil cuando no hay huracán imagínate cuando hay huracán.”** [*“I didn’t leave, well because, because I had a job and my husband had a job, but I wanted to leave, every day I said, “I’m going, I don’t want to be going through this.” Because I sometimes say, right, the infrastructure here is overwhelmed. You go to other countries and have these same difficulties, but the improvement is faster, faster. Sometimes I say, here it is like that, I don’t know if we live in a very strategic area for hurricanes, and it breaks us down the middle, but it is very difficult, how to go up? If it is difficult when there is no hurricane, imagine when there is a hurricane.*”] *No. of mentions (%): 3 (1.1%)*	**“Era con tratamiento, otros se fueron porque no tenían trabajo, pero la mayoría se fueron por tratamiento. Porque aquí los tratamientos para tu condición no estaban disponibles en ese momento. Y después pues, después de un par de meses, pues volvieron, pero se tuvieron que ir.”** [“*It was with treatment, others left because they did not have a job, but the majority left for treatment. Because here the treatments for your condition were not available at that time. And then well, after a couple of months, well they came back, but they had to go.”*] *No. of mentions (%): 5 (0.5%)*	**“En el caso mío, pues lo que me motivó a venir acá fue más bien la incertidumbre, cada vez yo no me sentía bien de salud, este, emocionalmente como él dijo, y también porque pues mi trabajo se afectó, y depende mucho de las comunicaciones, cuando me vine acá, pues obviamente mi mama me acompaña y ahí es que ella se queda que no pudo regresar a Puerto Rico y ahí fue que comenzamos a hacer la vida acá. He regresado, ido a Puerto Rico en varias ocasiones ya, este, porque tengo personas allá que obviamente trabajan conmigo y necesitan del apoyo mío, este, he visto como se ha ido recuperando poco a poco, pero básicamente pues ha sido bien difícil esa ayuda.”** [*In my case, because what motivated me to come here was rather the uncertainty, every time I did not feel good health, this, emotionally as he said, and also because my work was affected, and it depends a lot on the communications, when I came here, because obviously my mother accompanies me and that’s where she stays that she couldn’t return to Puerto Rico and that’s when we started living here. I have returned, gone to Puerto Rico on several occasions already, this one, because I have people there who obviously work with me and need my support, this one, I have seen how he has been recovering little by little, but basically, that help has been very difficult.”]* *No. of mentions (%): 48 (9.3%)*

Many participants report a total collapse of essential services such as electricity, water, and communication systems (internet and cellphone service). Those participants living in coastal and rural areas reported feeling secluded and experienced power outages for almost 1 year after Hurricane María.

From a participant living in a rural, coastal area:

“*aquí lo que más afectó fue pues, al no haber electricidad, fue el agua potable… pues, ocasionó bastantes problemas.”*

“here what affected the most was, since there was no electricity, it was drinking water ... well, it caused quite a few problems.”

From a participant living in a rural area:

“*ni agua, y ya va casi un año ahora, se cumple un año del huracán. Eso sí que es triste”*

“No water, and it’s been almost a year now, it’s a year since the hurricane. That is sad”

From a participant living in a rural area:

“*Ya yo había pasado por la experiencia de George. Cuando George yo vivía en San Juan. Y esa experiencia fue espantosa o sea y pasé por esa experiencia. Nosotros a los 3 meses ya nos habíamos recuperado de George, no fue como ahora que pasaron todavía son ocho meses y hay gente que no tienen, que están llenos de toldo y no tienen, hasta luz, hay personas que todavía no tienen electricidad.”*

“I had already been through George’s experience. When George came to San Juan. And that experience was horrible, I mean, I went through that experience. At 3 months we had already recovered from George, it was not like now that 8 months have passed and there are people who do not have, who are full of awning and do not have, even light, there are people who still do not have electricity.”

From a participant living in metro San Juan:

“*Fueron días de levantarse temprano, muy temprano, fueron días de quizá no levantarme temprano, de levantarme a cualquier hora, ir a darle rewind, quizás a las tres de la mañana va a haber señal, quizás a las 5 de la mañana va a haber señal, quizás a las 11 de la noche va a haber señal, y tú seguías llamando, tú seguías cargando el celular y tratando de llamar.”*

“There were days of getting up early, very early, there were days of maybe not getting up early, getting up at any time, going to give him a rewind, maybe at three in the morning there will be a signal, maybe at 5 in the morning there will be signal, maybe at 11 at night there will be a signal, and you kept calling, you kept carrying the cell phone and trying to call.”

The failure of essential services in the archipelago resulted, as described, in long waiting lines to access services such as food, water, and money.

From a participant living in a coastal area:

“*Lo que quería decirte era que creé un oído biónico para helicóptero. Cuando yo oía ese helicóptero de lejos, ya yo.. el oido biónico. Eso fue grande, tu sabes, correr al parque a buscar comida, wao correr al parque, esperar que un helicóptero te trajera comida. En una película es que se da eso. Y lo hicieron mucho, todos los días venían a traernos muchas cosas, un helicóptero a traernos comida.”*

“What I wanted to tell you was that I created a bionic helicopter ear. When I heard that helicopter from afar, I already ... the bionic ear. That was great, you know, running to the park to get food, wow running to the park, waiting for a helicopter to bring you food. In a movie, that happens. And they did a lot, every day they came to bring us many things, a helicopter to bring us food.”

From a participant living in a metro area:

“*Y entonces al otro día pues como “Estas bien” “Estoy bien” entonces pues comenzamos como había mencionado, desayuno, almuerzo, cena, este…..el cash, la gasolina, las filas, a la hora de dormir cuando llegaba la noche para dormir eso era horrible.*”

“And then the next day as “Are you okay” “I’m fine” then we started as mentioned, breakfast, lunch, dinner, this ... the cash, the gas, the lines, at bedtime when night came to sleep that was horrible.”

From a participant living in a metro area:

“*esa situación que todos vivimos en esos primeros días, desesperante. Si vas a sacar chavos obviamente la ATH no funcionaban y las que funcionaban las filas eran… kilométricas. O sea, era el problema de que tú ibas echar gasolina, tenías que hacer una fila que ibas a estar 3–4 horas, pero tenías que hacer 2–3 horas del día para sacar chavos de la ATH. Todo lo que aquí te tomaba en un día, recuerdo unos quince minutos. Eh, o sea que todo el día le podías dedicar a eso nada más. Así que comenzando por ahí era bien desesperante.”*

“That situation that we all lived in those first days, desperate. If you are going to get money, obviously the ATM did not work and the ones that worked the lines were ... kilometers. I mean, it was the problem that you were going to fill up gasoline, you had to stand in a line that was going to be 3–4 h, but you had to do 2–3 h a day to get money out of the ATM. Everything that took you here in 1 day, I remember about 15 minutes. Hey, so you could spend all day just that. So starting there was really maddening.”

Some participants described feeling “hopeless” and “scared” (Individual-level variables) in the time after the hurricane struck since they could not seek help or communicate with others in other parts of the island. Participants from these areas described how the debris and collapse of bridges made it impossible for them to seek outside help. Most participants believe that the lack of services combined with limited resources and unequal distribution of resources (e.g., food and water) resulted in the high crime rate in different parts of the island.

From a participant living in a rural area:

“*Porque yo misma estuve 50 días sin luz y a mí nadie fue a llevarme un pote de agua.”*

“Because I myself, was without electricity for 50 days and nobody came to give me a bottle of water.”

From a participant now living in Orlando, Florida:

“*Entonces cuando llego con mi tarjetita que fui con lo que uno se acostumbra a usar, pues no, tienes que pagar en efectivo, no esto es efectivo, todo es en efectivo y no tenía ni el efectivo ni teníamos para comer y pasamos un hambre terrible, eh, muchas veces así sin poder caminar llegaba a los lugares para buscar agua y cuando ya estábamos a punto de llegar al lugar donde estaba el agua, ya nos gritaban “se acabó, ya no hay más””*

“When I arrived with my card I went with it what you are used to use, well no, you have to pay in cash, this is not cash, everything is cash and I had neither the cash nor we had to eat and we were terribly hungry. Many times, without being able to walk I would arrive at the places to look for water and when we were about to reach the place where the water was, they would yell at us “it’s over, there is no more””

From a participant from an outer island:

“*Sí, muchas veces porque también hace poco empezaron a asaltar este los negocios. Se han llevado a varios presos, pero asaltaron los negocios, se llevaban mercancías, si quedaba dinero se lo llevaban.”*

“Yes, many times because also recently they began to storm this business. They have taken several prisoners, but they robbed the businesses, they took merchandise, if there was money they took it.”

While participants sometimes described feeling abandoned by both local and the federal government, this response was particularly noted in the coastal areas. Individuals reported that initial assistance was among community members: clearing debris, opening roads, and finding water and food. Coastal and rural participants described the assistance arriving mainly from two non-governmental organizations and volunteer workers from different community-level organizations.

From a participant living in a coastal area:

“*Bueno, yo por, en mi casa había un banco de, todo lo comida y los primeros, pues, cosas de primeros auxilios todo eso estaba en mi casa guardado, en un cuarto, verdad. Reservábamos un cuarto ahí, y había una lista de personas encamadas. A esos se les daba, siempre se les daban, siempre se les llevaban el agua, comida, pampers, chops, este, todo eso se les llevaba. Casi siempre…La mayor parte de la gente aquí siempre velaba por los vecinos de que estuvieran bien los que estaban encamados.”*

“Well, for me, in my house there was a storage of food, first aid things, all of that was stored in my house, in a room tight. We reserved a room there, and there was a list of bedridden people. They [food] were given to those, we always give them, they were always given water, food, pampers, chops, this, all of it. Almost always … Most of the people here always watched over their neighbors to make sure that those who were bedridden were well.”

From a participant living on an outer island:

“*Muchacha gracias a Dios a la Marina que la Marina trajo cajas de raciones que ni botándolas se acaban. Troces y troces y troces.”*

“Girl thank God to the Navy that the Navy brought boxes of rations that even throwing them out. Pieces and pieces and pieces.”

Some participants described that - amidst the circumstances concerning the aftermath of Hurricane María – some positive experiences (largely at the Community level) were of importance: for example, communities got together to help each other – participants described that in the aftermath of Hurricane María community members assisted each other (removing debris, sharing power generators), and communities were spending time with each other for the first time (playing card games, meeting each other, sharing food).

From a participant living in a rural area:

“*Si supieras... yo que estoy de presidente de comunidad desde enero de este año 2018 y lo que he visto, al contrario, había una desunión y ahora la comunidad se ha unido más, y a través de esta situación, de los problemas del agua y todas esas cosas, ha habido gente que no se hablaba y ahora se hablan, y esto ha traído, como que, ante las adversidades han salido cosas nuevas y buenas.”*

“If you knew … I have been the president of the community since January 2018 and what I have seen, on the contrary, there was a disunity and now the community has become more united, and through this situation, of the water problems and all those things, there have been people who did not speak to each other, and this has brought, like, in the face of adversity new and good things have come out.”

While the least common category mentioned in the ecological framework was “health care,” participants (particularly in rural areas) described challenges finding medications to manage pre-existing health conditions including such as diabetes and blood pressure. Additionally, individuals described challenges to access medical treatments including dialysis, chemotherapy treatment, as a result of medical systems collapse. Florida participants described that seeking medical care was sometimes a motivation for leaving. Participants in general, described emotional strains as the primary adverse health outcomes.

From a participant now living in Orlando, Florida:

“*En cuestión de hospital, mi pueblo perdió el único hospital que tenía. Nosotros no teníamos hospital. Así que, si ocurría una emergencia, tenía que correr la persona que estuviese conmigo, o con cualquier otra persona, para Guayama que son dos pueblos más allá o para Humacao, pero estaban cerrados, lo que había era uno nada más, era el Domínguez*.”

“When it comes to hospital, my town lost the only hospital it had. We didn’t have a hospital. So, if an emergency occurred the person who was with me, or with anyone else, had to run to Guayama, which are two towns away, or to Humacao, but they were closed, there was just one, it was the Dominguez.”

From a participant living in a coastal area:

“El daño más grande que ha hecho el paso de María a los sobrevivientes es el daño emocional que ha dejado en la isla. Me explico. El cual no sabía trabajar con ese daño emocional, ¿por qué? Volvemos a lo mismo, la persona que se afectaron emocional, son personas de tercera edad y niños. ¿Qué pasa? Que. aquí hay muy pocos psicólogos, pero esas personas que no vienen a hospitals.”

“The greatest damage that María’s passage has done to the survivors is the emotional damage it has left on the island. I explain. Who did not know how to work with that emotional damage, why? We return to the same thing, the person who was emotionally affected, are the elderly and children. What happens? That there are very few psychologists here, but those people who don’t come to hospitals.”

The main factors described influencing the decision to leave to the United States included: need of medical assistance, loss of property, losing employment, and the fear related to uncertainty based on the aftermath. Furthermore, participants living in Orlando, described that their main reason was related to overall better quality of life (e.g., the school for their children, lower crime rates). In contrast, some participants described challenges concerning financial limitations and the ability to seek medical care.

From a participant now living in Orlando, Florida:

*“Sí, lo mío fue más bien, yo como perdí mi carro este, me quedé sin trabajo, ahí conseguí lo de, lo de (nombre institución) que era temporal, pero luego de eso este…decidí moverme, y si voy a empezar desde cero, como muchas cosas, pues decidí hacerlo acá porque pienso que aquí hay bastantes oportunidades, pero, de hecho, si yo no hubiera perdido mi carro y mi trabajo, yo hubiera seguido en Puerto Rico, totalmente.”*

*“*Yes, mine was rather, since I lost my car, I was left without a job, that’s where I got the one from the (institution name) that was temporary, but after that … I decided to move, and if I go to start from scratch, like many things, because I decided to do it here because I think there are plenty of opportunities here, but, in fact, if I had not lost my car and my job, I would have remained in Puerto Rico, totally.”

From a participant living in a coastal area:

*“Mi hijo me envió el pasaje porque me tuvo que sacar. Por que yo soy de las que estaba en la calle y a mí me había dado un derrame cerebral completo y estaba en recuperación. Tuvieron que sacarme porque del asma no podía respirar ya. Tenía vecinos con generadores Diesel y le instalaron unos tubos bien alto para sacarlos de su casa el humo.”*

“My son sent me the ticket because he has to take me out. Because I am one of those who was on the street and I had a complete stroke and was in recovery. They had to take me out because of the asthma I couldn’t breathe anymore. He had neighbors with diesel generators, and they installed some pipes high up to get the smoke out of this house.”

## Discussion

Hurricane María (and Hurricane Irma before it) was a traumatic event that shifted the social, physical, and biological ecosystems of Puerto Rico. The hurricanes struck a social and physical landscape that, beforehand, was characterized largely by the social situations found in communities at that time (including crime and poverty, but also unity and social action), by hurricane preparation (or lack thereof) in households to protect material assets and to provide for the family’s food and water needs, and by individual sociocultural notions around perception of risk, spiritual protection, and a general underestimation of what was about to come. The traumatic period of when the hurricane struck and traversed the islands shifted focus to an intensely personal experience: most sentiments expressed in this period related to factors at the individual level, largely anxiety and fear. Similarly, the domains of Biological (e.g., anxiety) and Abiotic factors (e.g., wind, sound, damage) – largely absent from narratives of the pre-hurricane period – dominated during this phase. In the post-hurricane phase, Community-level and largely Sociocultural concerns dominated once again but with additional substantial attention to the Abiotic impacts of the hurricanes on the physical environment (built and natural) and to biological and health care factors that surrounded lasting anxiety and depression, new infectious diseases, and maintenance of medical treatments.

**Fig. 5:**
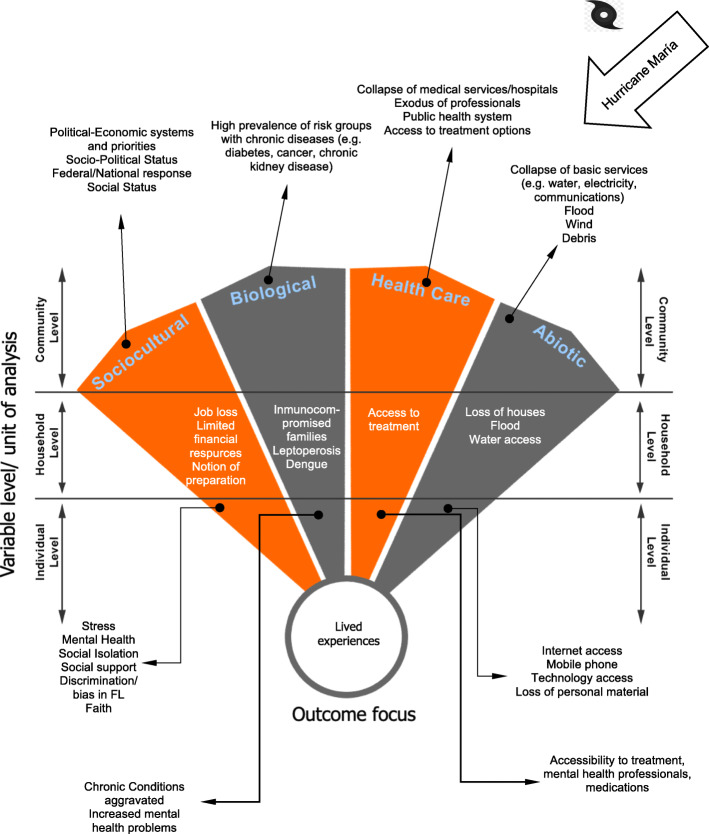
Critical Medical Ecological Model* Applied to identify salient issues related to Hurricane María. *adapted from McElroy and Townsend 2015

Figure 5 summarizes the constellation of salient ecological factors and constructs that emerged in this research. Previously presented, most codes were applied in the post-hurricane time period, most were applied to the sociocultural domain, and at the community level. The hurricane itself directly created biological reactions in individuals (for example fear, anxiety, and depression) along with the destruction (and resulting impacts) of the physical man-made and natural landscapes. New risks and conditions arose after the hurricane strike that may have been biological (e.g., leptospirosis, no food or water) or abiotic (e.g., roads and bridges washed away, structures destroyed) in nature, but created further, repeated, ongoing stressors and social needs for communities. As we found, concomitant with the physical landscape starting to recover and regenerate, the dynamics of the social and household landscape frequently involved the decision to leave Puerto Rico altogether, or forced people to continually face and adapt to an ongoing collapse in basic services that were only slowly and differentially restored. While Puerto Rico’s colonial situation created population migration for many years, the devastation caused by Hurricane María prompted many to move to the continental United States [9, 29]. Factors such as medical urgency, schools closing, and job loss were among the main reasons for leaving Puerto Rico among participants. This Puerto Rican exodus has been explored in multiple studies, pointing that most individuals migrated for reasons related to education, medical needs, and necessity for access to water and electric infrastructure [30, 31].

Such seismic shifts in entire ecosystems where landscapes and communities are completely altered as happened with the 2017 hurricanes in Puerto Rico are transformative stressors, forcing these landscapes and communities to respond and adapt in order to survive [32]. The ability of communities and the people living within them to respond and adapt rests, in large part, in the context of the disaster to begin with, and in their ability to mobilize assistance internally and externally to the community itself.

We found that factors that directly and indirectly related to Puerto Rico’s relationship with the USA were frequently mentioned in our interviews, often as a complicating factor in trying to respond to the emergency. Indeed, the limiting, defining factor in much of what happened in Puerto Rico before the hurricanes – and in accessing resources after them – is Puerto Rico’s on-going, colonial relationship with the United States [33, 34], which underscored the entire disaster and its components, and which made visible decades of social-environmental injustices, disparities, and inequities. Social and community-level conditions that pre-existed the hurricanes and their household and individual impacts were clearly and frequently mentioned by participants in this study. Prior to 2017 hurricane season, Puerto Rico already endured financial and humanitarian crises, diminished employment opportunities, an old and collapsing set of social and physical infrastructures (e.g., health care, electricity, water, communication), and widespread disparities and inequities within the population [35, 36]. The sluggish and often absent aid response from the United States delayed and prevented the ability of the population to adapt to a radically reshaped world in all regards [37, 38]. A rapidly declining population size post-hurricane [9] also left fewer people to help provide medical, social, and environmental assistance where needed, and destruction of the physical environment eliminated or stalled ecosystem services provided to the islands and communities of Puerto Rico (e.g., wildlife, crops, aquifers) [39].

The process of human and community adaptation to an acute stressor in the short term is one rife with sickness, tragedy, and chaos as people and systems redefine and rebuild in order to survive [40]. In Puerto Rico, for example, with thousands of deaths attributable to this ecological event [41], a shortage of health professionals [42], lack of electricity, water, communication, and government response [43, 44], and the inability to seek help outside of the communities [38], has created psychological and physiological stress and disease as a result [45, 46]. Several studies reported an increased risk for anxiety, depression, suicide, and PTSD [47–49]. From our study and others, the ongoing health effect of Hurricane María remains palpable in many communities of Puerto Rico and the diaspora in Florida [45, 50, 51]. In fact, some “adaptations” that help people adapt to one situation – for example self-medication in response to chronic stress [52], dietary shifts to calorie-dense, prepared foods [53], and exploitive forms of income generation [54, 55] – could well unintentionally create disease or challenges (a “mismatch”) in another [56]. The cumulative impact of these types of short-term cultural adaptations to environmental change could more or less permanently become embedded in a community over time, creating new, ongoing diseases not previously present nor predominant [32, 40]. This context of rapid, unstable change as individuals and communities struggle to adapt to a dramatically new reality in order to survive lasts long after the acute event that triggered the ecosystem change in the first place, and can have lasting, generational consequences.

Our study is limited by a restricted sample size, which could potentially not reflect the overall lived experience of people who were living in Puerto Rico at the time of the 2017 hurricanes. Our method prioritizes recall and vocalization of experience, frequently in the group discussion context of others; some participants may refrain from sharing their full experiences, and some may relay only partial experiences. We address these challenges through deliberate sampling across the country, with mixed individual and group interviews, and with an interview guide that encourages participants to describe their thoughts fully. Finally, using the Critical Medical Ecological Model as a framework assures we attend to all levels of variables across domains, including historical power dynamics.

Human communities constantly adapt to their shifting environments, and adapt their environments to accommodate their priorities. These processes create ongoing health and social effects – both intended and unintended – and communities entangled within dramatic, disastrous climate-induced events are particularly shocked and disrupted. As our data and others shows, individuals and their communities react and rebuild in response, frequently arising from local knowledge and insight, from building a community kitchen [57], medical and cleaning brigades, to other local community-organized recovery efforts [58].

That said, communities disrupted by ecological disaster are also entangled within global economic and political histories and dependencies which often preclude and prevent full recovery with minimal consequence in populations. Island nations are especially vulnerable to both climate-induced ecological change [59–61] and political-economic exploitation [62–64], yet their people have often developed and sustained resistant and cultural structures as a result of their exposure to these vulnerabilities [65]. Planning and preparing for ecologically-driven acute disasters in island environments need to account for the additive impacts of these relationships while considering simultaneously rapid ecosystem interdependent change among individuals, households, and communities.

## Supplementary Information

**Additional file 1.**

## Data Availability

All data generated or analyzed during this study are included in this published article. The qualitative interviews are not publicly available because of the risk of identification of participants, and of the risk of stigmatization of marginalized groups and communities represented by our research participants.

## Abbreviations

CDC: Centers for Disease Control and Prevention
CME: Critical Medical Ecological Framework
CITI: Collaborative Institutional Training Initiative
COREQ: Consolidated Center for Reporting Qualitative Research
PTSD: Post traumatic stress disorder
#p2p4PUR: people-to-people for Puerto Rico
