# Supplementary material for: “Y no quedó nada, nada de la casa, todo salió volando” (And there was nothing left, nothing of the house, everything flew away): a critical medical ecological perspective on the lived experience of hurricane María in Puerto Rico
Source: BMC Public Health. 2021 Oct 9;21:1833. doi: 10.1186/s12889-021-11847-w (PMC8502391; doi:10.1186/s12889-021-11847-w)
Supplement: Supplementary file 1 — Additional file 1. [file 12889_2021_11847_MOESM1_ESM.pdf]

Child code	Grandchild code	Description
**1. Comunidad**		
1.a Positivo	1.a1.Unión comunitaria	Participantes describen a su comunidad como una unida.
	1.b2 Tranquilidad	Participantes describen su comunidad como una tranquila.
1.b Negativo	1.b1 Job Security	Participantes describen las dificultades de conseguir trabajo en la comunidad.
2. Situacion Sociales		La crisis económica, la situación fiscal y social.
2.a Crímenes/violencia		Algunas personas reportan que estaban cansados de la criminalidad
		-"ya la situación de la criminalidad nos estaba incomodando."
2.b Falta de empleo		La situación económica en PR afectaba el ámbito laboral en los individuos.
2.c Muchas personas se estaban yendo de la Isla		Se habla de la cantidad de personas que se han ido de la isla debido a la dificultad económica.
3. Preparación para el huracán		Participantes relatan como se prepararon para el huracan
3.a Utilizar la preparación de Irma para el Huracán María		Participantes reportaron usar los productos comprados para Irma.
		Otros reportaron que habían dejado las tormenteras puestas desde Irma.
3.b Agua, comida enlatada para varios días o semanas.		La preparación consistió en comprar agua, comida enlatada, baterías que durara hasta dos semanas.
3.c Cubrir las ventanas y puertas con tormenteras/paneles		Participantes reportar que en sus preparativos incluyo cubrir las ventanas y puertas del hogar.
3.d Personas acudieron a refugios o la casa de algún familiar		Participantes reportaron que decidieron irse a otro lugar porque no encontraba su hogar seguro.
4. Falta de preparación ante la llegada del huracán		Hubo un consenso entre participantes donde mencionaron la falta de preparación para eventos catastroficos.
4.a falta de prevención y mantenimiento de parte de las agencias gubernamentales		Existia una falta de mantenimiento por parte de los municipios y el gob estatal. Ya sea en el recogido de escombros, basura, el tendido electrico, alcantarillas y carreteras
4.b Putting "preparation" on people who don't have the resources and are already struggling		Participantes reportaron que era dificil prepararse para el huracan con la situacion enconomica. "No todo el mundo podia cubrir los gastos"
5. Sense of invincibility – nothing will happen to us		La sobre confianza que existía en la comunidad porque no habían ocurrido eventos similares en mucho tiempo
5.a El efecto de sobreconfianza magnificado por Irma		Los participantes relatan que la seguridad de que el huracan Maria no iba a tocar PR se magnifico con el huracan Irma.
5.b "El cuento del lobo"		Referencia a que se decian que venian los huracanes y nunca llegaban.
		Es parte de la sobreconfianza
		Participantes relatan que su poca preparacion se debio a que no creian que el huracan iba a llegar. "ay, yo no voy a comprar nada, para que hacer tanta fila si eso no viene, a última hora dicen que eso se va a desviar"
6. Underestimated what was coming/ didn't expect or anticipate the level of destruction		"Nadie esperaba el impacto ni el nivel de destruccion" Participantes relatan que era imposible prepararse para la magnitud de el evento.
7. Initial hit of Irma made the destruction of Maria worse		Algunos participantes reportaron que los daños ocurridos por el huracan Irma (I.e. falta de luz) agravaron la situacion ante Maria.
8. Aferrarse a la Fé- como herramiento para que no pasara nada		El uso de la religion (fe) como herramienta para creer que el huracan no llegaria.
		"Dios nos va a proteger"
8.a"Puerto Rico es una isla bendecida"		Las personas utilizaban este decir para hablar sobre como estariamos bien y que no nos iba a pasar nada.
9. Relatos/Experiencia		Experiencias vividas durante el huracán
9.a Relatos sobre el viento		e.g. "Era un monstruo, el ruido del huracán era un monstruo."
		"El viento retumbaba la casa"
9.b Relatos de experiencias (en general) durante el huracán		Participantes relatan sus experiencias durante el huracan. Que hicieron, como la pasaron, y si problemas que enfrentaron.
**10. Miedo y ansiedad durante el huracán**		Participantes relatan la desesperación que sentían en medio del huracán. Sentían miedo y ansiosos por qué no sabían cuando se iba a terminar.
10.a "Dios que esto pare ya"		
10.b Falta de comunicación en medio del huracán		Participantes relatan el desespero de no saber que estaba ocurriendo en medio del huracán cuando toda comunicación cesó.

10.c Anxiety of people not in Puerto Rico unsure about the		Participantes relatan que la falta de comunicacion ocasiono desesperacion en aquellos que viven fuera de PR al no saber de sus familiares
11. Uso de los medios sociales (Fb, twitter, whatsApp).		Las personas durante la tormenta trataron de usar las redes sociales para saber que ocurría, pero la falta de conexión imposibilitaba el uso.
12. 12. Relatos sobre las experiencias en los primeros dias		Participantes relatan sus experiencias en los primeros dias luego de el huracan
12.a "Parecia que una bomba callo y secó todo"		Participantes relatan su impresion al salir de sus hogares luego de el huracan.
12.b "Los vecinos se chequearon entre ellos para asegurarse que todos esten bien"		Participantes relatan que tan pronto el huracan ceso, los vecinos se "chequearon" entre ellos para asegurarse que todos estan bien.
13. Relatos de trauma para la comunidad		Durante y Después del huracán, por ejemplo, inundaciones, incertidumbre por falta de comunicación/electricidad, miedos.
14. Experiencias vividas en la comunidad (+)		
14.a "Los niños jugaban en la calle y la gente se hablaban entre si"		Participantes relatan que algo positivo de esta experiencia es poder volver ver a los niños jugando afuera y volver a la union comunitaria donde la gente compartian juntos y comenzaron a conocerce.
14.b Los miembros de la comunidad se unieron para abrir paso y limpiar		Relatos de como la comunidad se unió para limpiar los escombros
14.c Los miembros de la comunidad se unieron para proveer ayuda entre ellos		Relatos sobre la comunidad ayudándose entre sí a buscar alimentos y agua para aquellos que no podían ir.
15. Experiencias vividas en la comunidad (-)		
15.a Indifference toward other people's situations		Relatos sobre miembros de la comunidad no ayudandose entre si.
		**"Nadie con planta se ofreció poner la insulina en la nevera"**
15.b Initially people were neighborly but as things improved, people started to go back to being closed off from others		Participantes relatan que los vecinos se ayudaron entre si al principio, pero luego que las cosas comenzaban a volver a la normalidad volvieron a individualisarse.
16. Ayuda/Auxilio		En general las personas hablaban de como tuvieron que pedir ayuda luego del huracán
16.a "Nos dejaron solos" después del huracán"		ayuda
16.a.1 Help wasn't coming		Participants expressed that the agencies left them alone. They felt that help from government agencies didn't arrived.
16.b Falta de ayuda entre miembros de la comunidad		Express the perception of not receiving help from community members.
		Situaciones donde no se ayudaron entre si
16.c Situaciones familiares que se agravaron luego del huracán		Relatos de problemas en las familias que se agravaron luego del huracan
16.d La gente que estaba enferma y no estaba recibiendo la ayuda		Hubo personas que comentaron sobre como escucharon o tuvieron personas en su comunidad que estaban enferman y la ayuda no les estaba llegando
17. Colapso de servicios básicos		
17.a El colapso de servicios básicos	17.a1 La falta de electricidad	Relatan como sobrevivieron ante la falta de electricidad y que hacían para llevar a cabo sus tareas diarias.
	17.a2 No Cash available	"La falta de dinero en efectivo. Los bancos estaban cerrados y luego te limitaban la cantidad de dinero que podias sacar"
	17.a3 Lines for buying gas, ice	"Las filas para buscar gasolina eran de horas y aveces no llegabas a coger nada"
	17.a4 La falta de alimentos	Participantes relatan la falta de alimentos en lo ssupermercados y las filas largas que se tenian que hacer para poder conseguirlos.
	17.a5 El crímen aumentó	Participantes relatan el alza en crimines en las noches. En especial robos.
	17.a6 La falta de agua	Participantes relatan los estragos antes la falta de agua. Buscaban agua en el rio o en los oasis.
17.b La falta de comunicación. "lo peor fue estar descomunicados, no habia manera de saber de	17b. La falta de comunicación. "lo peor fue estar descomunicados, no	Participants expressed concern due to the lack of communication and desperation because they were not able to reach/communicate with their people.
		Preocupación por no poder comunicarse ni saber las condiciones de sus conocidos.

nadie"	17.b1 El caos generado por no tener comunicación en general	Participantes relatan "el caos" que ocurrio por la falta de comunicación y medio informativos.
	17.b2 Loss of mobile phone service and internet	Relates to the failure of cellphone service.
		Hace alusión al fallo del servicio telefónico celular.
	17.b3 Las personas manejaban a otros pueblos para conseguir señal	Las personas que tenian que ir a lugares de altura o al area metropolitana buscando señal de celular.
18. El rol de la "media"		
18.a La gente en los EEUU sabian mas de lo que habia pasado que los que lo vivieron	18.a La gente en los EEUU sabian mas de lo que habia pasado que los que lo vivieron	Personas describen como "the media" jugo una funcion en sus vidas y en los de aquellos que vivian fuera de PR.
18.b Use of social media (FB, WA) to learn about what was happening.	18.b Use of social media (FB, WA) to learn about what was happening.	Este Código refleja el uso de los medios sociales para saber que estaba ocurriendo. Ya sea aquellos dentro de PR o fuera de PR buscando familiares.
**19. Incertidumbre de no saber de los familiares dentro de la isla.**		Este código refleja la frustración que existía entre miembros de la familia que aún residiendo en Puerto Rico quedaron incomunicados.
**20. Falta de acceso a servicios de salud**		Mucha gente reportó como los problemas de salud en su comunidad (diabéticos, diálisis) se agravaron por la falta de acceso.
20.a Condiciones de salud en general se agravaron		Participantes relatan que las condiciones de salud se agravaron por la falta de medicamentos, electricidad, agua, acceso a hospitales.
		e.g. manejo de diabetes, alta presión, pacientes en dialisis
20.b Leptosperosis		"Me preocupaba al escuchar los casos de la gente que se estaba muriendo por estar tomando agua. Habia escuchado de que el orín del ratón causaba daño"
		"Ay, yo no me arriesgué, yo no me atreví, porque como estaba lo de la cuestión esta, de que había orines en el agua, (E: ¿de ratón?) De ratón entonces pues, yo sé que a veces la gente, pues, que vende la comida, a veces no tienen buena higiene, y como no había luz, no había agua, pues me daba manía, pues trataba de cocinar lo más posible."
20.c Colapso de servicios hospitalarios		Participantes relatan que la falta de electricidad causo un cierre en la mayoría de los hospitales. Las condiciones detrimentes de los hospitales aumentaron las enfermedades.
20.d Falta de medicamentos en los hospitales		Participantes relata que los hospitales/farmacias tenían una escasez de suministros.
20.e Escasez de médicos		Participantes relatan que la deficiencia de medicos en la isla se agravo luego del huracan. No habían médicos suficientes para lidiar con la situación.
20.f Problemas de Salud Mental		
	20.f1Trauma	Participantes relatan traumas relacionados al paso del huracán. "La lluvia nos pone nervioso"
	20.f2 PTSD/triggers	
	20.f3 Problemas para dormir	Participantes relatan tener dificultades para dormir luego del paso del huracán.
	20.f4 Falta de ayuda para lidiar con el post-trauma	Participantes relatan que debido a la limite comunicación que hubo por meses, existía una falta de ayuda para lidiar con el post-trauma.
	20.f5 Suicide risks and narratives	"…y a base de la depresión viene el suicidio, y se escucharon muchos casos en Puerto Rico de personas no solo que se enfermaron, también optaron, la tomaron es decisión de suicidarse, (HF3: del desespero) el desespero que había…"
	20.f6 El impacto hacia la salud mental el irse solos a EEUU	La salud mental de estar alejado de tu país. "Que dejaste todo allá, que lo perdiste todo. Tus sueños se perdieron"
**21. Coping strategies for the aftermath (narrative)**		Relatos sobre las estrategias de afrontamiento que utilizaron luego del huracán.
		"Lo más cercano a una ayuda mental, fue esto" (refiriéndose a este estudio)"
21.a Uso del alcohol, y el humor, como un "coping mechanism"		Algunos participantes relatan el uso del humor y la fiesta entre vecinos como una estrategia de afrontamiento.
22.a Carretera bloqueadas (debris)		Relatos sobre carreteras bloqueadas por arboles caidos, postes, tendido electrico, etc.
22.b Estructuras perdidas		Relatos sobre perdida de hogares o edificios
22.c Inundaciones		Relatos sobre la inundacoines en la comunidad.
22.d Perdida de empleo		Participantes relatan la perdida de empleo luego de el huracan Maria.
22.e Incremento en la presencia de mosquitos		Las personas no podían dormir tranquilos por la cantidad de mosquitos en al área. Especialmente por la falta de electricidad o techo seguro, las personas estaban más expuestas a estos problemas.

22.f Incremento en la presencia de roedores		
22.g Problemas de agua potable		Participantes relatan la falta de agua potable y que hacian para conseguirla.
	22.g1Buscaban agua de los rios	Participantes relatan que obtenian agua de los rios para el aseo personal.
	22.g2Buscaban agua en los oasis	Participantes relatanq ue obtenian agua delos oasis para uso diario/aseo personal.
**23. AID/ RELIEF**		Participantes describen los tipos de ayudas que recibieron.
23.a Institutions and large organizations delivering aid and assistance		Participantes describen los tipos de ayudas que recibieron de diferentes organizaciones gubernamentales.
23.b La ayuda venia de familiares viviendo en el exterior		Participantes relatan que la ayuda que recibían venia de familiares viviendo en el exterior.
23.c Relatos de las personas realizando trabajo voluntario para ayudar dentro y fuera de la comunidad		Participantes describen sus experiencias haciendo trabajo voluntario.
23.d Gratitude of people receiving help		Participantes describen como se sintieron cuando les llegaba la ayuda.
23.e La gente no se queria comer la comida que estaban dando (MRE's)		Algunos Participantes no les gustaba la comida que daban de emergencia.
23.f La ayuda solo llegaba algunas partes		Las personas hablaban de que las ayudas llegaban al centro del pueblo pero aquellas comunidades que quedaban distantes no podian o no llegaban a tiempo para poder recibirlas.
24. Maneras de adaptación ante la falta de servicios		Participantes relatan que estrategias usaron para adaptarse ante la falta de servicios basicos.
25. Fé "conexión espiritual"-RELIGIÓN		Las personas se aferraron a la religión como una manera para lidiar con las dificultades asociadas a el paso de María.
26. Death narrative (story)		Participantes relatan la acumulación de muertos en la morgue, muertes de familiares o vecinos.
26.a Opiniones relacionadas a las muertes		Participantes relatan sus opiniones acerca de el tema de las muertes durante/despues el Huracan Maria
**27. Injury narrative (story)**		Relatos sobre heridas/lesiones que ocurrieron luego del paso del huracán.
**28. Recomendaciones de la comunidad para capacitación en el futuro**		Capacitaciones de manejo de emergencias y ayuda a nivel comunitario para no depender de las agencias gubernamentales. Participantes piensan que se puede "educar" a las personas para la preparación de huracanes desde un nivel comunitario.
**29. Lo que se aprendió de este evento**		Participantes relatan las cosas que aprendieron desde el paso del huracan.
**30. Relocalización a EEUU**		
**30.a Experiencias en general relacionadas con irse de PR**		Participantes relatan cómo se sintieron cuando se fueron de PR y los obstáculos que tuvieron que enfrentar.
		"Devastador, ese primer mes, esos primeros así fue el primero, los primeros días era—como dijo aquí el caballero—era irreconocible oscuridad. Aunque fuera de día era oscuro porque había mucho sol, pero uno sentía la soledad y la desgracia, se sentía en el aire, lo sentíamos muchos."
		"experiencia agridulce porque uno sabía que estaba dejando mucha gente atrás"
	30.a1Dificultades para conseguir trabajos	Participantes relatan lo difícil que se les hizo conseguir trabajo.
**30.b Relatos de decidir irse para EEUU**		En general como las personas relataron como fue esa decisión de irse para EEUU
	30.b1 La diferencia de tener a tu familia en EEUU como instrumento de apoyo	Participantes relatan como tener un soporte en EEUU sirvio de apoyo
	30.b2 El irse sin tener a nadie en EEUU	Participantes relatan sus experiencias de tener que irse sin tener a nadie.
	30.b3 Deseo de mantener una conexión con la patria desde el exterior.	Este sentimiento de regresar al país pero realmente solo como visita. "Estar aquí y allá"
	30.b4 The dream among diaspora of returning to Puerto Rico	Participantes relatan sus deseos de volver a la isla.
**31. Relatos de vivir en los hoteles**		Participantes relatan sus experiencias viviendo en los hoteles.
		"Lo que nos vendieron no era"

31.aEl no querer estar los hoteles por que causaba angustia		Participantes expresaban que a veces estar en los hoteles era algo parecido a estar en una prision.
31.b El no tener el espacio para hacer sus cosas		Participantes relatan que en los hiteles no habia espacio/privacidad para hacer sus cosas.
31.cEl tener que estar mudandose de hoteles o habitaciones		Participantes relatan sus experiencias de tener que estar mudandose constantemente de cuartos.
31.d Situación de no saber que pasaria cada vez que se anunciaba que FEMA quitaria las ayudas		Participantes relatan la angustia de no saber que iban a hacer si quitaban las ayudas.
32. Social Disparities		
32.a Awareness (or lack of it) of pre-existing disparities in Puerto Rico		Participantes relatan que el paso del huracan les dio a conocer o destapo los problemas sociales que existen en PR. “María nos enseño la otra cara de la pobreza en Puerto Rico”
33. El racismo y discrimen que se vive en la Florida		Participantes relatan los retos que viven en cuanto a la discriminación racial; incluyendo entre latinos mismos.
34. El acceso a la salud en EEUU en comparación con Puerto Rico		Participantes relatan sus experiencias positivas en el acceso a la salud en EEUU
		E.g., El no tener que esperar tanto para que te vea un médico
35. El acceso a la salud en EEUU en comparación con Puerto Rico (-		Participantes relatan sus experiencias negativas en el acceso a la salud en EEUU.
		E.g. El costo asociado de ser atendido en los EEUU. Los relatos de ir a sala de urgencia
36. Relatos del porque se quieren quedar en EEUU		Participantes relatan por que se quieren quedar en EEUU
36.a Acceso a mas oportunidades de trabajo		La gente hablaba de en comparación con Puerto Rico habian mas oportunidades de trabajo en EEUU. También que se podían mover a otros estados si era necesario.
36.b “Se siente uno mas Seguro”		Las personas relataban que se sentian mas seguros en EEUU que en PUR.
36.c Mejor educación para los niños		Participantes relatan que una de las razones que se quieren quedar en EEUU es porque sus hijos reciben mejor educación.
36.d Wants to return		Participantes explican sus deseos de volver a PR
36.e Wants to return, but cannot due to hardship		Participantes relatan que desean volver a PR pero no pueden debido a los problemas sociales que enfrenta
36.f Wants to return, but cannot due to medical reasons		Participantes relatan que desean volver a PR pero no pueden debido a los problemas de salud
37. azones por la cual no consideraron irse del país		Participantes describieron su rol en quedarse en la isla luego del huracán. Pensaban que tenían que quedarse para poder “echar la isla para adelante”.
		“Era un deber patriótico”
38. Relatos de regresar a PR luego de que pasará la tormenta		Participantes relatan sus deseos de regresar a PR/ o como regresaron a PR luego que las cosas comenzaron a mejorar.
39. Reshaping landscape		Participantes relatan como su entorno cambio luego del paso del huracán.
		Las casas y vecindarios que antes no se veían”
**40. Issues impacting pregnant women**		Eventos que hayan ocurrido con relación a las mujeres embarazadas, ya sea en antes, durante y despues del huracan
**41. Issues impacting children**		An infant had a choking episode due to using a different formula thank the one he was used too.
		Niño murió electrocutado al jugar en una caja eléctrica que no se sabía que estaba "viva" (energizada).
**42. Impact of hurricanes on Puerto Rican identity (narratives)**		Relatos sobre como el huracan influencio la identidad de los puertorriqueños
**Ao Stressors brought up by participants**		Ejemplos de estresores que los participantse describieron en cuanto a los obstaculos luego de el huracan
**43. Age Wisdom (diferencia entre generaciones**		Como el haber vivido pasados huracanes/pasadas experiencias (e.g. no celulares) les permitio saber como manejar la situacion comparado con las generaciones mas jovenes.
**44. Exemplary narrative (analytic code)**		Historias significativas sobre las experiencias durante/despues de el Huracan Maria.
**45. Medical ecology narrative**		Historias que definan las experiencias durante/después de el huracán Maria que reflejen el modelo ecológico.
**46. Interesting quote (analytic code)**		Añadir quotes interesantes o importantes para analisis en el futuro
